# Nanobodies targeting ABCC3 for immunotargeted applications in glioblastoma

**DOI:** 10.1038/s41598-022-27161-3

**Published:** 2022-12-30

**Authors:** Eduardo Ruiz-López, Ivana Jovčevska, Ruth González-Gómez, Héctor Tejero, Fátima Al-Shahrour, Serge Muyldermans, Alberto J. Schuhmacher

**Affiliations:** 1 Molecular Oncology Group, Instituto de Investigación Sanitaria Aragón (IIS Aragón), 50009 Zaragoza, Spain; 2 Center for Functional Genomics and Biochips, Institute of Biochemistry and Molecular Genetics, Faculty of Medicine, University of Ljubljana, Ljubljana, Slovenia; 3 Bioinformatics Unit, Centro Nacional de Investigaciones Oncológicas (CNIO), 28029 Madrid, Spain; 4 Cellular and Molecular Immunology, Vrije Universiteit Brussel, Pleinlaan 2, 1050 Brussels, Belgium; 5 Fundación Aragonesa para la Investigación y el Desarrollo (ARAID), 50018 Zaragoza, Spain

**Keywords:** CNS cancer, Biologics, Antibody fragment therapy

## Abstract

The cancer “omics” reveal many clinically relevant alterations that are transforming the molecular characterization of glioblastomas. However, many of these findings are not yet translated into clinical practice due, in part, to the lack of non-invasive biomarkers and the limitations imposed by the blood–brain barrier. Nanobodies, camelid single-domain antibody fragments, emerge as a promising tool for immunotargeted applications for diagnosing and treating glioblastomas. Performing agnostic bioinformatic analysis from glioblastoma patient datasets, we identified ATP Binding Cassette subfamily C member 3 (ABCC3) as a suitable target for immunotargeted applications. The expression of *ABCC3* is associated with poor survival and impaired response to temozolomide. Importantly, high expression of *ABCC3* is restricted to glioblastoma, with negligible levels in healthy brain tissue, and further correlates with tumor grade and stemness markers. We identified three immunogenic epitopes of ABCC3 which were used to isolate nanobodies from a glioblastoma-specific phage-display nanobody library. Two nanobodies targeting ABCC3 (NbA42 and NbA213) were further characterized and demonstrated in vivo selective recognition of ABCC3 in glioblastoma xenograft mouse models upon systemic administration. We designate NbA42 and NbA213 as new candidates to implement immunotargeted applications guiding a more personalized and precise diagnosis, monitoring, and treatment of glioblastoma patients.

## Introduction

Glioblastoma is a highly aggressive and heterogeneous primary brain tumor. According to the WHO central nervous system (CNS) 5 classification, glioblastoma is an *Isocitrate Dehydrogenase* (*NADP*( +)) (IDH)-wildtype diffuse and astrocytic grade 4 glioma in adults which presents microvascular proliferation or necrosis or mutation of *Telomerase Reverse Transcriptase* (*TERT*) promoter or amplification of *Epidermal Growth Factor Receptor* (*EGFR*) gene or changes in + 7/ − 10 chromosome copy number^1^. Advances in molecular biology and "omic" sciences (e.g., transcriptomics, genomics, metagenomics, epigenomics, proteomics, metabolomics) provide clinically relevant biomarkers for the further development of powerful tools to study the biology of glioblastoma and to identify novel therapies^2^.

Despite advances in the molecular classification of glioblastoma, there is an urgent need to designate novel molecular targets to improve patient management strategies. Overall survival has not changed in past decades and remains at 14.6 months for patients with primary glioblastoma. This life expectancy is reduced to 6.7 months after relapse^3,4^. The current standard of care of glioblastoma patients includes maximal surgical resection, followed by radiotherapy with concomitantly and adjuvant chemotherapy with the alkylating agent temozolomide (TMZ)^3,4^. TMZ is an alkylating prodrug that delivers a methyl group to purine bases of DNA generates O^6^-methylguanine in DNA, a cytotoxic lesion removed by O^6^-methylguanine DNA methyltransferase (MGMT). Methylation of the *MGMT* promoter results in gene silencing and correlates with better outcome in TMZ-treated glioblastoma patients^5^. Although a tissue sample from surgery or biopsies is often required, genetic alterations and epigenetic modifications have uncovered their relevance towards more personalized treatment strategies. The combination of *IDH1* mutations and *MGMT* promoter methylation status offers a higher predictive potential of survival in glioblastoma patients than either alone^6^.

A hurdle in diagnosing, monitoring, and treating glioblastoma is imposed by the blood–brain barrier (BBB) by limiting the effectiveness of most of the strategies against this tumor. This highly selective filter restricts access from the bloodstream into the brain parenchyma for almost 100% for large molecules and more than 98% for all small-molecule drugs targeting the CNS^7^. Molecules showing both lipophilic solubility (formation of less than eight hydrogen bonds with solvent water) and MW < 400 Da may be transported through the BBB via lipid-mediated free diffusion^8^. Although drug reengineering contributes to BBB transcytosis, pharmacologically significant amounts of many CNS targeting drugs are unreachable, leading to treatment failure. The range of access of proteins in the blood to the CNS is only 0.01–0.4%, including antibodies with intrinsic therapeutic potential^9^. A wide variety of smaller protein scaffold-based drugs is being developed to circumvent BBB selectivity^10^.

Nanobodies represent an innovative alternative due to their reduced size (12–15 kDa; 2.5 nm × 4 nm), constituting one of the smallest molecules with intact functional antigen-recognition capacity^11^. Nanobodies are single-domain antigen-recognizing fragments from the heavy-chain-only antibodies of *Camelidae*. They contain three Complementarity Determining Regions (CDRs) with, on average, larger hypervariable H1 and H3 antigen-binding loops than human antibodies^12^. These characteristics enable nanobodies to recognize unique conformational epitopes, such as cavities or unstructured regions of proteins, with high affinity and stability^13,14^. Besides, various molecular mechanisms for delivering nanobodies through the BBB have been extensively described. Nanobodies targeting CNS molecules have been characterized to cross the BBB by receptor-mediated transcytosis and adsorptive-mediated transcytosis. In this regard, nanobodies have also been utilized not only as shuttle platforms for modeling BBB-permeable drugs but they also have served to improve the targeting of multifunctional therapies (e.g., based on nanoparticles) into the brain parenchyma^15,16^.

Performing agnostic bioinformatic analysis in multiple datasets of glioblastoma patients, we identified differentially expressed genes coding for proteins located in the plasma membrane. Among them, we selected ATP-Binding Cassette subfamily C member 3 (ABCC3, also known as MRP3) as a biomarker of glioblastoma, suitable for further development of immunotargeted applications with clinical impact. The ABC transporter superfamily comprises the largest family of proteins for translocating substrates across membranes^17^. Nine of the ABCC subfamily members are designated multidrug resistance proteins (MRPs), as they mediate cancer multidrug resistance (MDR) by actively extruding chemotherapeutic agents out from tumor cells, constituting targets for cancer therapy^18^. Among them, ABCC3 is an organic anion transporter responsible for effluxing anticancer compounds^19,20^. ABCC3 has been involved in chemotherapy failure and reduced survival in various cancers^21,22^. Also, several reports have elucidated a plausible role of ABCC3 in the pathology and prognosis of glioblastoma patients^23,24^. ABCC3 may constitute a promising target for future molecularly targeted clinical intervention of glioblastoma.

We have isolated several nanobodies targeting ABCC3 by following a peptide-based strategy for biopanning of a previously constructed glioblastoma-specific phage-displayed library^25^. Five of these nanobodies showed in vitro specific binding of ABCC3 transporter expressed on the cell surface. Two nanobodies, NbA42 and NbA213, were further characterized and demonstrated selective recognition of ABCC3 transporter in vivo upon systemic administration in glioblastoma xenograft mouse models. Therefore, we propose that NbA42 and NbA213 could serve as molecular candidates for developing immunotargeted applications that could improve the diagnosis, monitoring and therapy of glioblastoma patients.

## Results

### ABCC3 is a target for glioblastoma immunotargeted applications

**Figure 1:**
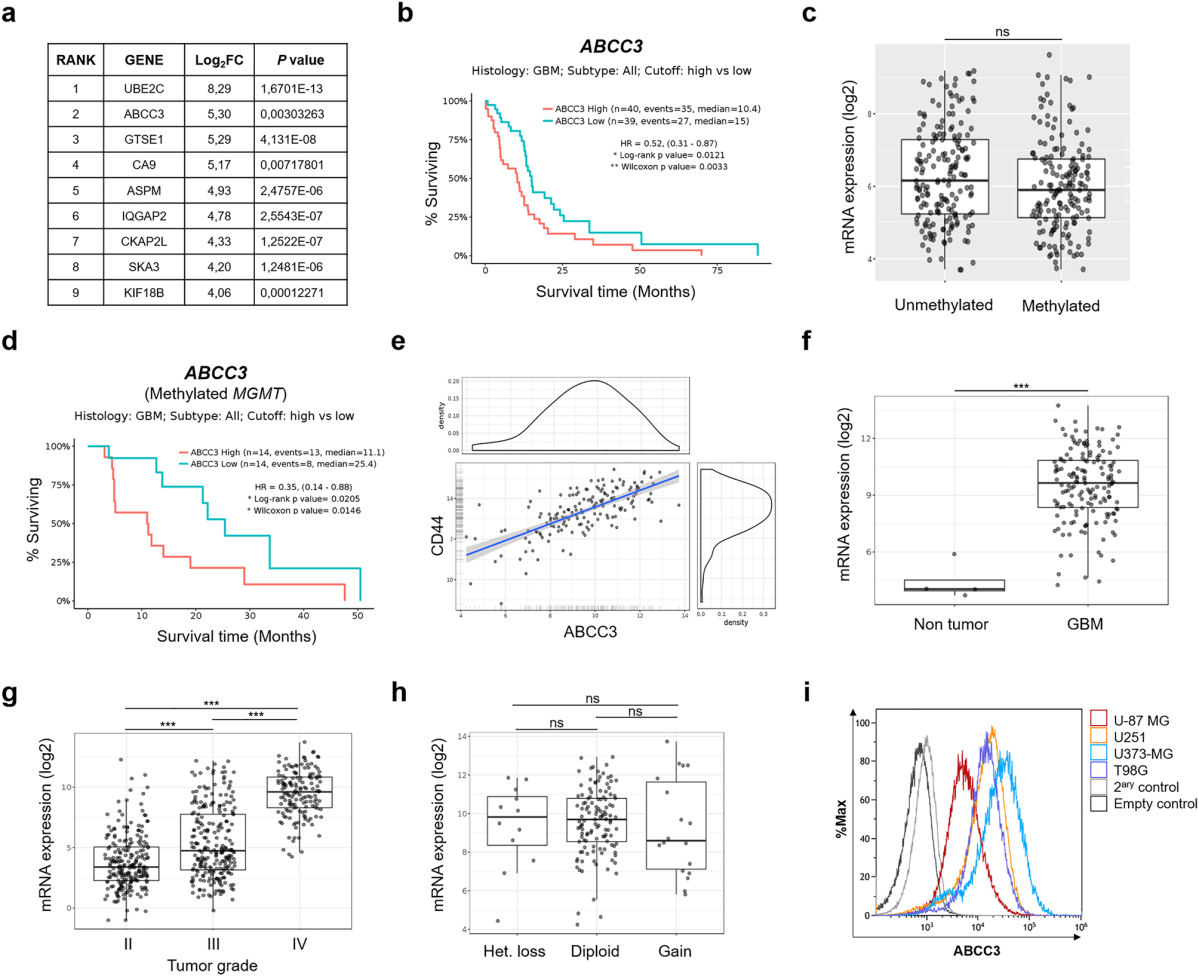
ABCC3 is highly expressed in glioblastoma and correlates with poor prognosis. (**a**) List of membrane-associated genes differentially expressed in glioblastoma vs. normal tissue (Log_2_FC > 4; data from TCGA, https://www.cancer.gov/tcga^26^, accessed on April 26th, 2017): *Ubiquitin Conjugating Enzyme E2 C* (*UBE2C*), *ATP Binding Cassette subfamily C member 3* (*ABCC3*), *G2 And S-Phase Expressed 1* (*GTSE1*), *carbonic anhydrase 9* (*CA9*), *Assembly Factor For Spindle Microtubules* (*ASPM*), *IQ Motif Containing GTPase Activating Protein 2* (*IQGAP2*), *Cytoskeleton Associated Protein 2 Like* (*CKAP2L*), *Spindle And Kinetochore Associated Complex Subunit 3* (*SKA3*), and *Kinesin Family Member 18B* (*KIF18B*). (**b**) Kaplan–Meier curves for overall survival in the TCGA-GBM dataset (RNA-Seq, GlioVis) for ABCC3*.* (**c**) *ABCC3* expression is independent of *MGMT* methylation status. Tukey's Honest Significant Difference (HSD). TCGA-GBM dataset (RNA-Seq, GlioVis). The figure shows the difference between pairs (mean ± SEM), the 95% confidence interval and the p-value of the pairwise comparisons; ns, non significant. (**d**) Kaplan–Meier curves for overall survival in the TCGA-GBM dataset (RNA-Seq, GlioVis) for *ABCC3* in *MGMT* methylated patients. Log-rank and Wilcoxon test for survival curves. Cut off top high vs low. **p < 0.01; *p < 0.05. (**e**) Correlation of *ABCC3* and *CD44* expression. Pearson's product-moment correlation. TCGA-GBM dataset (RNAseq, GlioVis). ***p < 0.001. (**f**) *ABCC3* expression in glioblastoma (n = 156) and control brain (n = 4) (ATCC-GBM dataset, RNA-Seq, GlioVis; mean ± SEM). (**g**) *ABCC3* expression increases with tumor grade (ATCC-GBMLGG dataset, RNA-Seq GlioVis; mean ± SEM). Grade II (n = 226), Grade III (n = 224), Grade IV (n = 150). (**h**) *ABCC3* expression and copy number alterations (CNA) from the ATCC-GBM dataset (RNA-Seq, GlioVis; mean ± SEM). Heterozygous loss (n = 12), Diploid (n = 121), Gain (n = 18). HSD test for (f), (g) and (h). ***p < 0.001; **p < 0.01; *p < 0.05; ns, (**i**) ABCC3 expression in U-87 MG, U251, U373-MG, and T98G glioblastoma cell lines determined by flow cytometry. Empty control consists of unstained cells.

Candidate targets of glioblastoma for the development of immunotargeted applications were selected based on (1) their higher gene expression on glioblastoma compared with healthy tissue, and (2) the location of the encoded protein on the extracellular surface of the plasma membrane. To identify the most differentially expressed genes in glioblastoma, we performed a bioinformatic analysis of 142 glioblastoma and 5 normal brain samples (RNA-Seq) deposited in The Cancer Genome Atlas (TCGA)^26^ using *limma* (v. 3.24.15, R package)^27^. According to these criteria, 287 genes (Supplementary Table S1) were identified and ranked by differential expression (Log_2_FC). 9 genes were identified to be the most differentially expressed in glioblastoma compared to healthy tissue (Log_2_FC > 4) encoding for membrane-associated proteins (Fig. 1a). Further analysis using GlioVis^28^ revealed that only the expression of *ABCC3* and *CA9* correlated with worse patient overall survival (Fig. 1b, and Supplementary Figs. S1a, S1d, S2). The role of CA9 in glioblastoma has been described^29^ and nanobodies against CA9 have been previously developed^30^. In addition, the implication of ABCC3 in drug resistance and stemness potential of a variety of tumors has been described^20^. Therefore, we further investigated the role of ABCC3 in glioblastoma.

Despite studies that have suggested higher *ABCC3* expression levels in *MGMT* methylated patient samples^20^, we found similar *ABCC3* levels of expression independently of the *MGMT* methylation status (Fig. 1c). Importantly, we found that higher expression of *ABCC3* in patients with methylated *MGMT* promoter correlates with a worse prognosis (Fig. 1d). A possible role for ABCC3 in TMZ resistance and cancer stemness could be hypothesized in glioblastoma. Importantly, we found a correlation between the expression of stemness markers (*CD44* and *FUT4* (*CD15*)) with *ABCC3* in glioblastoma (Fig. 1e, and Supplementary Fig. S1c,d,h,i). *ABCC3* presents lower expression levels in normal brain than in glioblastoma samples (Fig. 1f, and Supplementary Fig. S3), and its expression increases with glioma tumor grade (Figs. 1g, and Supplementary Fig. S1b,e). *ABCC3* is located on human chromosome 17 and its expression in glioblastoma is independent of copy number alterations (Fig. 1h) suggesting that *ABCC3* expression is a regulated process during glioblastoma development instead of a consequence of chromosomal instability (e.g., duplication, deletion, or *loci* amplification). We also observe high levels of expression of ABCC3 in a panel of glioblastoma cell lines (Fig. 1i). These data point to ABCC3 as a suitable molecular target that meets the requirements for further developing nanobody-based immunotargeted tools for glioblastoma.

#### Selection of three immunogenic epitopes of ABCC3 as suitable targets

**Figure 2:**
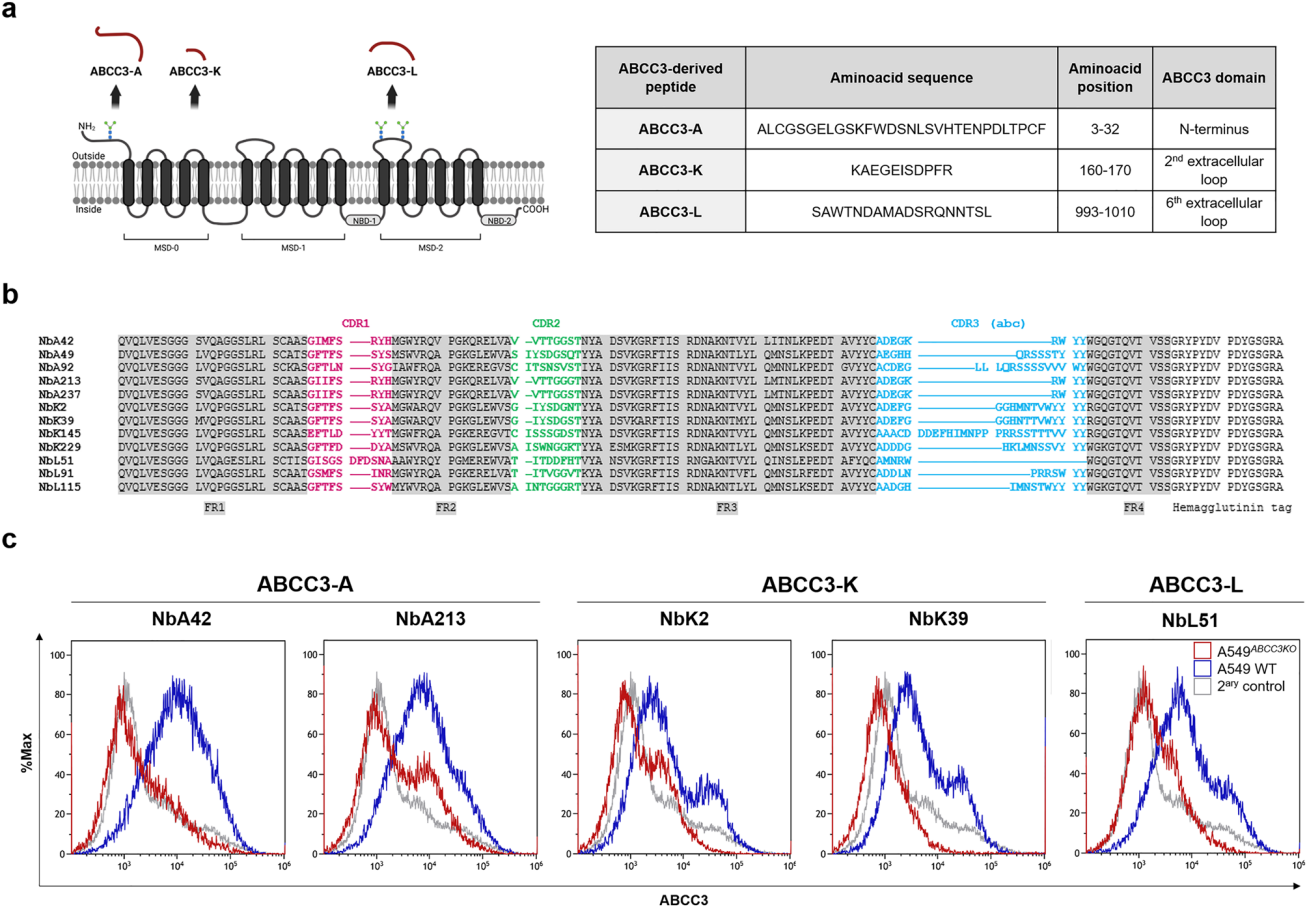
Identification of nanobodies targeting ABCC3 following a peptide-based strategy for biopanning. (**a**) Proposed membrane topology of ABCC3 (UniProtKB reference: O15438). ABCC3 comprises two Membrane Spanning Domains (MSD-1 and MSD-2), each of them containing 6 transmembrane helices and followed by one Nucleotide-Binding Domain (NBD-1 and NBD-2), and an additional N-terminal domain containing 5 transmembrane helices (MSD-0). Extracellular N-glycosylation sites may regulate substrate specificity, stability, and localization of ABCC3^19^. The location and amino acid sequence of the selected peptides in extracellular regions utilized to isolate specific nanobodies targeting ABCC3 from a phage display library is shown. (**b**) Amino acid sequence of the isolated nanobodies targeting ABCC3 depicting their Framework Regions (FR1-4, grey) and Complementarity Determining Regions (CDR1, pink; CDR2, green; and CDR3, given in alphabetic order, blue). Nanobodies NbA49, NbK2, NbK39, NbK229 and NbL115 present a VH-like VHH imprint in their framework 2 region (GLEW sequence). Nanobody NbL51 presents an extremely short CDR3 domain, which the longer CDR1 loop might compensate for, and a modified GLEW motif with a three-amino acid insertion. Some nanobodies targeting ABCC3 have an R118 residue instead of W118 as the first amino acid of the framework 4 region, which is exclusive of VHHs and not presented in humans, mouse nor camel VH. (**c**) Screening of the immunoreactivity of nanobodies targeting ABCC3 by flow cytometry. The immunoreactivity of 2 nanobodies targeting peptide ABCC3-A (NbA42, NbA213), 2 nanobodies targeting peptide ABCC3-K (NbK2, NbK39), and 1 nanobody targeting peptide ABCC3-L (NbL51) was confirmed. A549 cell line (A549 WT) and its ABCC3 knocked-out derivative cell line (A549^*ABCC3KO*^) were used as positive and negative controls. Secondary staining controls are also shown.

A glioblastoma-specific phage-display library of nanobodies with ~ 1 × 10^8^ transformants was previously constructed^25^. Screening and selection of nanobodies targeting ABCC3 were performed following a peptide-based strategy. We predicted immunogenic epitopes within the amino acidic sequence of ABCC3 (UniProtKB reference: O15438) using BepiPred-2.0 analysis software^31^. Candidate immunogenic epitopes were selected based on (1) their sequential nature, as linear B-cell epitopes are commonly used for immunizations and antibody production^32^, (2) their length, with limitation to a minimum of 11 and a maximum of 30 amino acids to approximate the size of current linear epitopes^33,34^, and (3) their outer surface location at the plasma membrane, restricted to extracellular regions of the Membrane Spanning Domains (MSDs) and N-terminus of ABCC3 (Fig. 2a). The bioinformatic analysis yielded 57 potential immunogenic epitopes (Supplementary Table S2), from which four peptides met these requirements. Their precise alignment with the ABCC3 sequence was confirmed utilizing the Basic Local Alignment Search Tool (BLAST) Protein (BlastP). As shown in Fig. 2a, one of the selected peptides (ABCC3-A, amino acids 3–32) is located in the N-terminus, while the other three (ABCC3-K, amino acids 160–170; ABCC3-V, amino acids 557–569; and ABCC3-L, amino acids 993–1010) are located within extracellular loops of MSDs of ABCC3. Three of them were synthesized with an addition of a 6× His-tag at the C-terminus (ABCC3-A and ABCC3-K) or N-terminus (ABCC3-L), for further screening of nanobodies.

#### Identification and screening of nanobodies targeting ABCC3

For nanobody enrichment, each ABCC3-derived peptide was used for three rounds of biopanning of the phage-display library^25^. We identified 12 nanobodies with different and complete sequences (Fig. 2b; Supplementary Table S3). We selected five nanobodies recognizing peptide ABCC3-A (NbA42, NbA49, NbA92, NbA213, NbA237), four nanobodies with ABCC3-K (NbK2, NbK39, NbK145, NbK229), and three nanobodies with ABCC3-L (NbL51, NbL91, NbL115). The selective targeting of the isolated nanobodies against ABCC3 was evaluated by flow cytometry (Supplementary Figure S4a). The human lung adenocarcinoma A549 cell line (A549 WT) presents the highest levels of ABCC3 expression (Human Protein Atlas, http://www.proteinatlas.org)^35^ and was selected as a positive control for the initial experiments. We knocked-out ABCC3 by CRISPR/Cas9 technology. The resulting cell line (A549^*ABCC3KO*^) was used as a negative control (Supplementary Fig. S4b).

Five nanobodies showed significant differential binding capacity (Fig. 2c). The immunoreactivity of two nanobodies targeting peptide ABCC3-A (NbA42, NbA213), two nanobodies targeting peptide ABCC3-K (NbK2, NbK39), and one nanobody targeting peptide ABCC3-L (NbL51) was confirmed. NbA42 and NbA213 were the nanobodies with the highest potential to detect ABCC3 in vitro and were further evaluated.

### Molecular characterization of anti-ABCC3 nanobodies

#### Nanobodies NbA42 and NbA213 specifically recognize ABCC3

**Figure 3:**
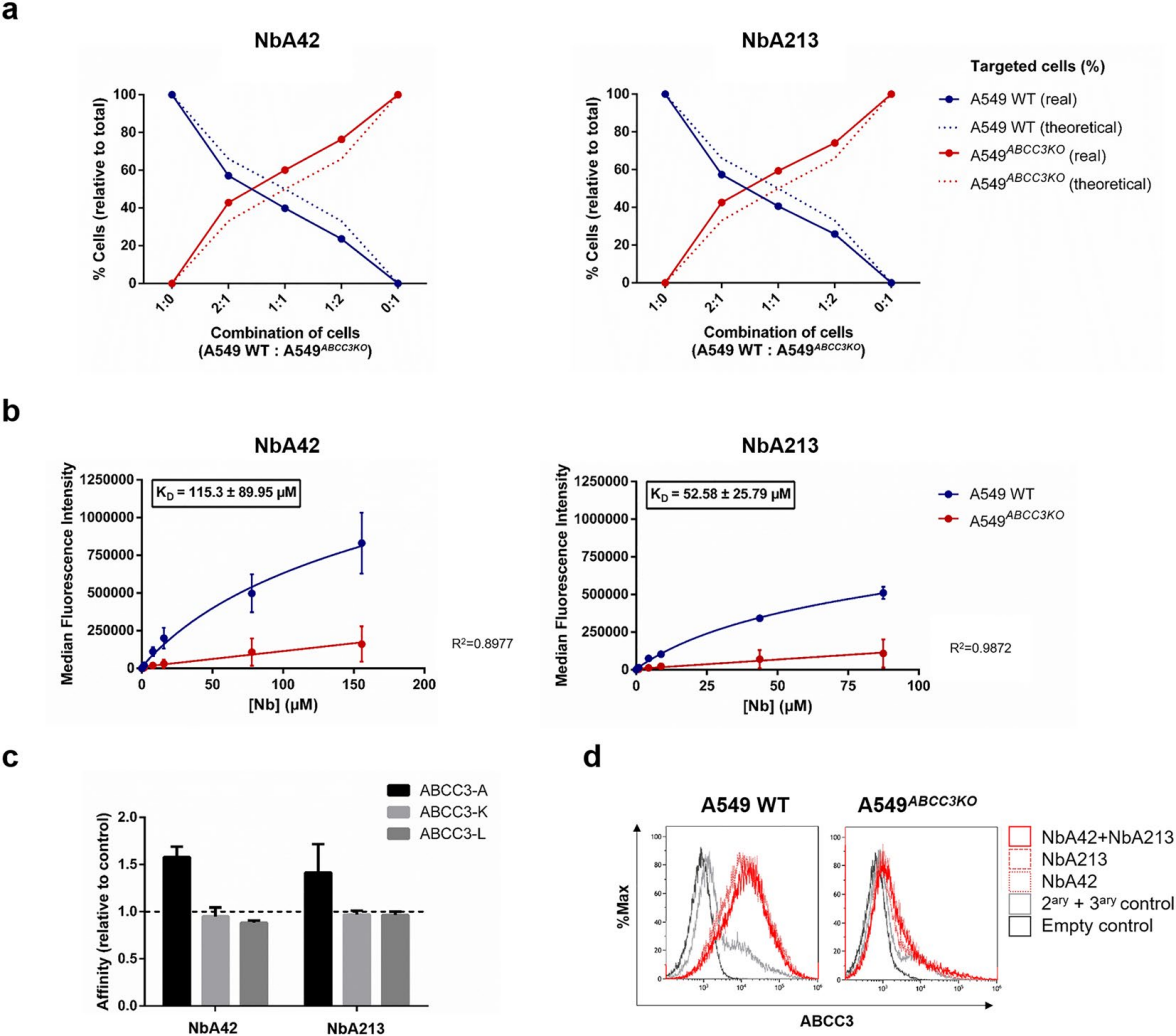
Evaluation of the targeting potential of NbA42 and NbA213 against ABCC3. (**a**) NbA42 and NbA213 specifically target ABCC3-expressing cells (A549 WT) over A549^*ABCC3KO*^ control cell line by flow cytometry. Both cell lines were combined at different proportions. Percentage of targeted cells, relative to the total number of cells, is shown for each proportion (X axis). Theoretical percentages of targeted cells, given by each ratio, have been included (dotted line). (**b**) Binding affinity of NbA42 and NbA213. Their equilibrium dissociation constant (K_D_) was calculated by flow cytometry (n = 2). Increasing concentrations of FITC-labeled nanobodies were used to quantify ABCC3 expression in A549 WT and A549^*ABCC3KO*^ control cell lines. (**c**) Binding specificity of NbA42 and NbA213. The selective binding to the ABCC3 peptide used for their isolation by biopanning (e.g., ABCC3-A, ABCC3-K and ABCC3-L) was calculated by indirect ELISA. The dark dotted line represents the background signal yielded by the negative control (0.1 M NaHCO_3_ coating buffer without peptide) (n = 2). (**d**) Dual-nanobody flow cytometry to evaluate synergies in the detection of ABCC3 in vitro.

The potential of targeting ABCC3 by NbA42 and NbA213 was evaluated by flow cytometry using ABCC3-expressing and loss-of-function control cell lines (Fig. 3a), demonstrating the potential to discriminate different proportions of these cell types in vitro. Subsequently, both nanobodies were labeled with fluorescein-5-isothiocyanate (FITC) to determine their equilibrium dissociation constant (K_D_). The affinity of NbA42 and NbA213 was assessed by targeting the control cell line A549 WT, using A549^*ABCC3KO*^ as a non-specific background control (Fig. 3b). Results showed that both nanobodies bind whole-length ABCC3 protein in vitro (K_D_ = 115.3 ± 89.95 µM for NbA42 and K_D_ = 52.58 ± 25.79 µM for NbA213). We confirmed the specific recognition of NbA42 and NbA213 to peptide ABCC3-A among the other ABCC3-derived peptides (e.g., ABCC3-K, and ABCC3-L) by indirect ELISA (Fig. 3c). The combination of NbA42 and NbA213 demonstrated no synergy to detect ABCC3 in vitro (Fig. 3d), further validating the common recognition of peptide ABCC3-A.

#### NbA42 and NbA213 can target ABCC3 in vivo

**Figure 4:**
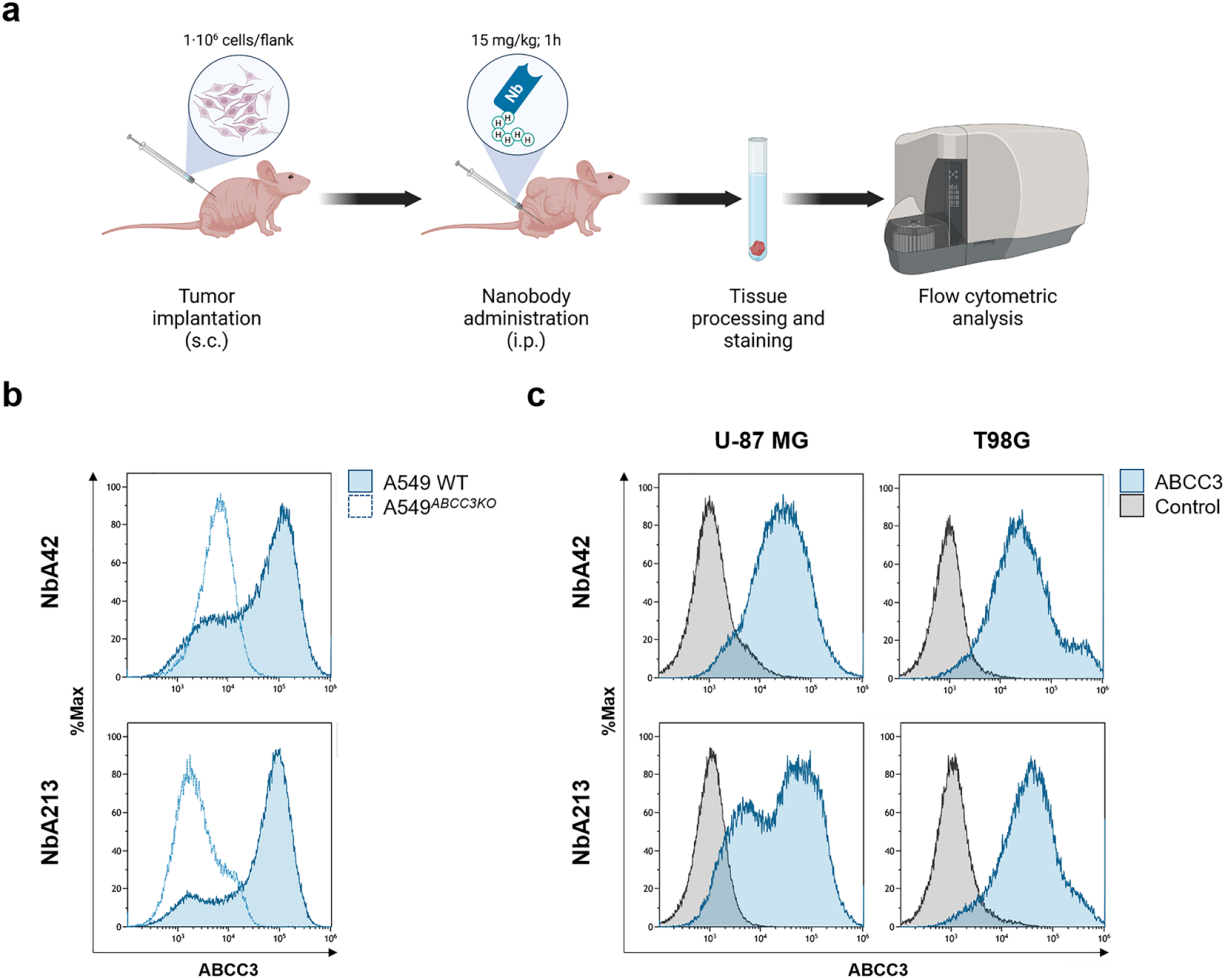
NbA42 and NbA213 recognize ABCC3-expressing tumors in vivo. (**a**) Workflow of the experimental setup of targeting analysis of nanobodies. Nanobodies were administered at 15 mg·kg^-1^ intraperitoneally (i.p.) in xenografted mice with the indicated cell lines, and tumor targeting was examined using prospective ex vivo flow cytometry, 1 h after systemic injection. Image created with BioRender.com (accessed on November 19th, 2021). (**b**) Evaluation of the in vivo targeting of NbA42 and NbA213 in ABCC3-expressing and loss-of-function cell lines (e.g., A549 WT, and A549^*ABCC3KO*^). (**c**) Assessment of the potential detection of ABCC3-expressing glioblastoma tumors by NbA42 and NbA213 in vivo.

The capacity of NbA42 and NbA213 to target ABCC3 in vivo was evaluated in xenografted tumor-bearing mice of the A549 and A549^*ABCC3KO*^ control cell lines. Nanobodies were administered intraperitoneally (i.p.) at 15 mg·kg^−1^ and their specific binding was analyzed 1 h after systemic injection (Fig. 4a). Similar to the in vitro data, systemic injection of NbA42 and NbA213 demonstrated selective detection of ABCC3 in vivo (Fig. 4b). Both nanobodies specifically bound A549 WT xenografted tumors (ABCC3-expressing cells), compared with the background of A549^*ABCC3KO*^ xenografted tumors (ABCC3-loss-of-function cells). NbA42 and NbA213 showed minimal residual signals in other tissues (Supplementary Fig. S5a). Accordingly to previously reported data on the pharmacokinetic profile of other nanobodies^36–38^ NbA42 and NbA213 were early detected in blood plasma after i.p. administration and cleared from circulation by renal clearance (Supplementary Fig. S5b). NbA42 and NbA213 were not detectable in plasma samples 4 h upon systemic administration.

**Figure 5:**
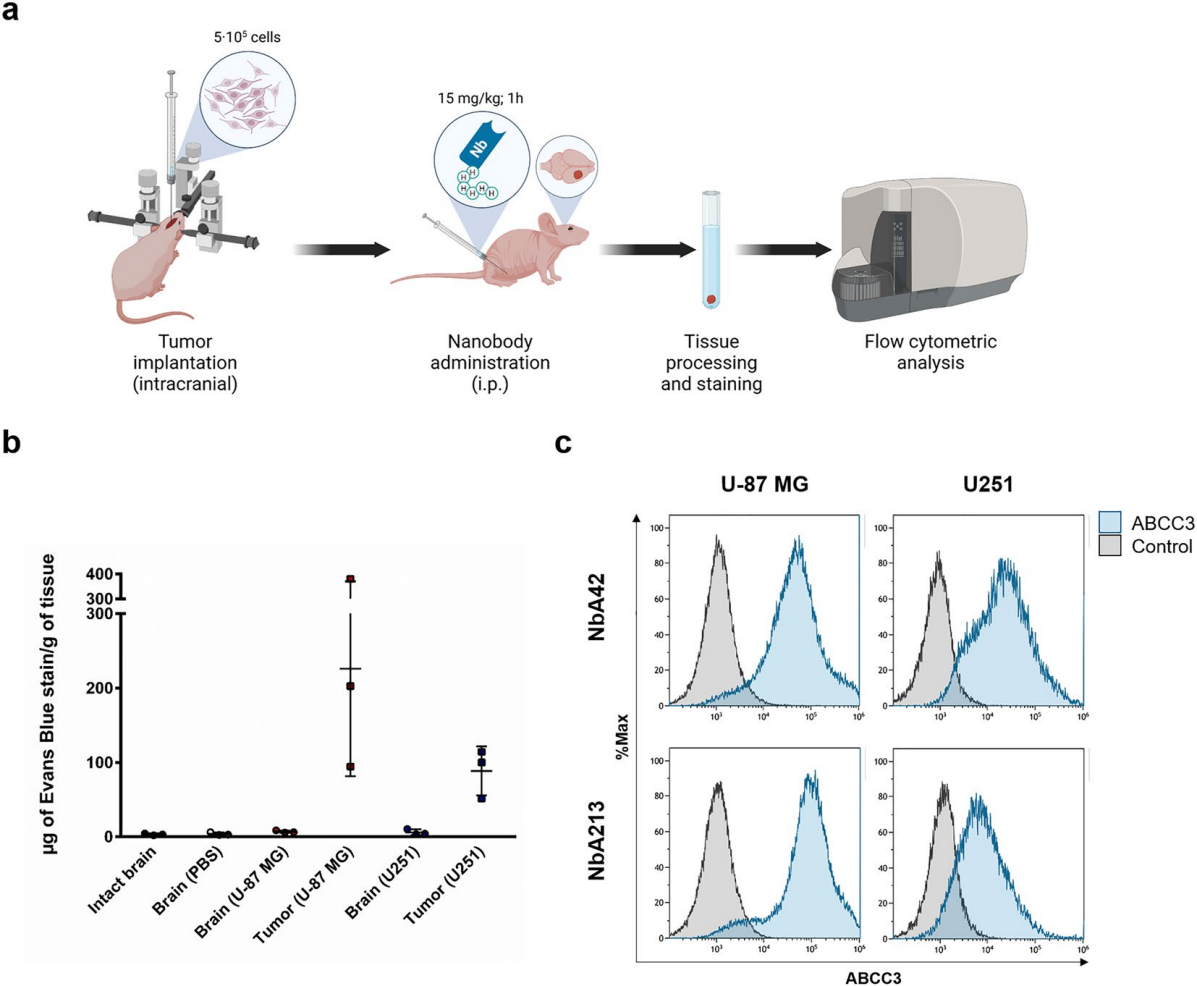
NbA42 and NbA213 recognize ABCC3-expressing orthotopic brain tumors. (**a**) Workflow of the experimental setup of targeting analysis of nanobodies. Nanobodies were administered at 15 mg·kg^−1^ i.p. in mice bearing intracranial glioblastoma tumors generated from the indicated cell lines. Tumor targeting was examined using prospective ex vivo flow cytometry, 1 h after systemic injection. Image created with BioRender.com (accessed on November 5th, 2022). (**b**) Assessment of the potential detection of ABCC3-expressing orthotopically implanted glioblastoma tumors by NbA42 and NbA213. (**c**) Analysis of BBB integrity by quantification of i.p. administered Evans blue in the brain of mice with intact, mock surgery brains (PBS), orthotopic U251 and U-87 MG tumors and their contralateral hemisphere. Graphic depict the amount of Evans blue stain per gram of tissue (mean ± SD; n = 3).

NbA42 and NbA213 also target ABCC3-expressing glioblastoma cell lines. Both nanobodies showed a similar in vitro targeting of ABCC3 in U-87 MG, U251 and T98G cells (Supplementary Fig. S6). We next evaluated the capacity of NbA42 and NbA213 to selectively target glioblastoma cells in vivo. First, we analyzed the selective targeting of NbA42 and NbA213 in U-87 MG and T98G heterotopic xenografted tumor-bearing mice in vivo (Fig. 4c). One hour after systemic administration, both nanobodies recognized ABCC3-expressing heterotopic xenografted glioblastoma tumors. We next evaluated the potential of NbA42 and NbA213 to detect ABCC3-expressing orthotopic brain tumors (Fig. 5a). Two orthotopic glioblastoma mouse models, with different degrees of BBB disruption, were utilized. As expected, U-87 MG derived tumors presented a higher degree of damaged integrity of the BBB than U251 derived tumors (Fig. 5b). Both, NbA42 and NbA213, detected either both U-87 MG and U251 orthotopic brain tumors (Fig. 5c) suggesting that these nanobodies does not present a high dependence for a highly disrupted BBB. Altogether, these results point to NbA42 and NbA213 as candidates for further development of immunotargeted applications for glioblastoma diagnosis and/or therapy.

## Discussion

"Omic" sciences hold the promise of precision medicine in cancer by translating molecular-based big data into novel clinical approaches^39^. Performing agnostic bioinformatic analysis across multiple datasets we identified ABCC3 as a suitable candidate to develop immunotargeted tools for glioblastoma. ABCC3 transporter is a member of the multidrug resistance proteins (MRPs) which confers resistance to various chemotherapeutic anticancer compounds^19^. In agreement with other reports we found that *ABCC3* is highly expressed in glioblastoma samples, correlates with tumor grade and worse prognosis of patients^23,24^.

ABCC3 may have a plausible role in temozolomide (TMZ) resistance. The expression of ABCC3 increases following TMZ treatment in glioblastoma cell lines, in a concentration and time-dependent manner^40,41^. Also, the specific inhibition of ABCC3 with MK571 increases apoptosis upon TMZ treatment in other cell types^42^. The higher expression of ABCC3 in NK cells modulates their chemoresistance to TMZ in patients of malignant gliomas^43^. Recently, *ABCC3* has been included in gene signatures (*ABCC3*, *CD44*, *TNFRSF1A*, and *MGMT*^24^; *ABCC3*, *SMC4*, *EMP3*, *WEE1*, and *HIST1H2BK*^44^) that classify glioma patients in agreement with therapeutic response to chemotherapeutic agents, including TMZ. According to previous reports^20^, we found higher *ABCC3* expression in patients with methylated *MGMT* promoter, correlating with a worse prognosis. A role of ABCC3 in cancer stemness is emerging, as *ABCC3* knockdown reduced the expression of stemness genes and the CD44^high^/CD24^low^ breast cancer stem-like subpopulation. Also, SOX2 mediates transcription of *ABCC3* in glioblastoma cells^45^. We found a similar correlation between *CD44*, *FUT4* (*CD15*) and *ABCC3* expression across glioblastoma patient datasets. These data support the possible intervention of ABCC3 in TMZ resistance and cancer stemness. Then, ABCC3 could represent a new molecular target of glioblastoma which may facilitate the subsequent development of molecular imaging approaches for patient management.

Novel immunotargeted applications obtained by combining the selectivity of antibody derivatives with the resolution capabilities of a given imaging modality, have been implemented for glioblastoma theragnosis. Single-chain variable fragments (scFvs) against ABCC3 have been used in preclinical models of glioblastoma. Interestingly, scFvs M25, M58, and M89 recognized extracellular epitopes of the N-terminus of ABCC3 and showed specific targeting of ABCC3-expressing glioblastoma cells in vitro^46^. However, the access of scFvs into the CNS is limited and fusion to cell-penetrating peptides acting as BBB shuttles is often required^47^. Nanobodies can circumvent the BBB by taking advantage of molecular routes of transcytosis^15,16^. In recent years, several nanobodies have sprouted against glioblastoma targets of protein biosynthesis (TUFM, TRIM28), DNA metabolism (NAP1L1), and cellular growth (EGFR, DPYSL2, β-Actin)^48–50^. Here, we describe two novel nanobodies targeting ABCC3, NbA42 and NbA213, isolated from a previously constructed glioblastoma-specific phage-display library^25^. The rational design of immunogenic-like peptides located on the extracellular surface of ABCC3 allowed a peptide-based strategy for biopanning. In vitro studies showed that NbA42 and NbA213 specifically target ABCC3 at the N-terminus (peptide ABCC3-A). NbA42 and NbA213 recognized ABCC3-expressing cells in glioblastoma heterotopic xenografted tumor-bearing mice. Detection of ABCC3 by NbA42 and NbA213 was further achieved in two orthotopic glioblastoma mouse models with different degrees of BBB disruption. While further characterization of the BBB penetrance is required, these results suggest that these nanobodies do not present a high dependence on a highly disrupted BBB. Future applications of both nanobodies targeting ABCC3 may allow the development of novel glioblastoma theragnostic procedures.

Nanobodies constitute a versatile tool for cancer management^51^. Among others, nanobodies have been exploited to deliver cytotoxic payloads specifically to tumors, image-guided surgery, and to improve chimeric antigen receptor (CAR)-T and photodynamic therapies^52–56^. Since the approval of nanobody ALX-0681 (Caplacizumab) by the regulatory agencies^57^, examples of radiolabeled nanobodies conducting immuno-positron emission tomography (immuno-PET)^58^ studies have reached clinical trials in cancer patients. Regarding glioblastoma, ^89^Zr-labeled nanobodies targeting HGF have demonstrated their theragnostic potential in preclinical models^59^. We highlight the potential of NbA42 and NbA213 as new candidate molecules for the further design of nanobody-based molecular imaging probes. The predictive role of ABCC3 in the survival and prognosis of glioblastoma patients reveals a newfangled translational diagnostic and/or therapeutic relevance of NbA42 and NbA213 in the clinics.

## Materials and methods

### Cell cultures

Human lung adenocarcinoma A549 and its ABCC3 knocked-out by CRISPR/Cas9 system derivative A549^*ABCC3KO*^ cell lines, and U-87 MG, U251, U373-MG, and T98G human glioma cell lines were grown with DMEM medium (Sigma-Aldrich) containing 10% FBS (Fisher Scientific), and maintained at 37 °C and 5% CO_2_. A549 and U373-MG were kindly provided by Dr. Martín-Duque (IACS). U-87 MG, U251, and T98G were kindly provided by the Holland and Joyce laboratories (MSKCC). Short tandem repeat markers performed cell line authentication at different passages.

### Bioinformatic analysis and statistics

RNA-Seq data from 142 glioblastoma samples and 5 normal brain samples deposited in The Cancer Genome Atlas (TCGA; https://www.cancer.gov/tcga accessed on April 26th, 2017)^26^ were analyzed using *limma* (v.3.24.15, R package)^27^ to identify the most highly expressed genes. The differentially expressed list of genes was mined for those that encode proteins located in the plasma membrane. The subcellular localization was determined by experimental immunohistochemistry evidence as cataloged in the Human Protein Atlas (http://www.proteinatlas.org^35^, “compartments” database^60^). Gene expression and survival of adult glioblastoma patient data from the TCGA Project (TCGA-GBM and TCGA-GBMLGG), the Chinese Glioma Genome Atlas (CGGA, http://www.cgga.org.cn/ accessed on October 5th, 2021)^61^, and the Repository for Molecular Brain Neoplasia Data (REMBRANDT, https://wiki.cancerimagingarchive.net/display/Public/REMBRANDT)^62^ were analyzed using GlioVis data portal (http://gliovis.bioinfo.cnio.es/)^28^. Log-rank and Wilcoxon tests were applied for Kaplan–Meier survival curves. Pearson's product-moment correlation was applied to determine gene correlations between paired samples. Tukey's Honest Significant Difference (HSD) was applied to compare *ABCC3* expression across tumor grades and glioblastoma vs. normal samples. Statistical analysis was obtained from GlioVis and completed using R (http://www.r-project.org).

### Biopanning of nanobodies

Prediction of immunogenic epitopes of human ABCC3 (UniProtKB reference: O15438) was performed by submitting the amino acidic sequence to BepiPred-2.0 analysis software (IEDB, https://www.iedb.org/ accessed on August 1st, 2018)^31^. A list of 57 potential epitopes was obtained (Supplementary Table S2) and manually curated for those located in the outer part of the plasma membrane. Three peptides, corresponding to immunogenic-like extracellular ABCC3 epitopes, were selected and His-tagged synthesized (Thermo Scientific):

- ABCC3-A (ALCGSGELGSKFWDSNLSVHTENPDLTPCFHHHHHH),
- ABCC3-K (KAEGEISDPFRHHHHHH),
- ABCC3-L (HHHHHHSAWTNDAMADSRQNNTSL).

These peptides were used for biopanning as described elsewhere^63^, using an existing phage-display library of nanobodies generated upon immunization of an adult male alpaca with whole patient-derived stem-like-enriched glioblastoma cells^25^. For each ABCC3-derived peptide, the library was subjected to 3 sequential rounds of biopanning by facing 1 µg peptide in 0.1 M NaHCO_3_, pre-coated on NUNC anti-histidine ELISA plates (Thermo Scientific), with 10^11^ phages for 1 h at room temperature. Non-specific binding sites were blocked with 5% nonfat milk. Bound phages were eluted with 100 mM triethylamine and neutralized with 1 M Tris–HCl (pH 7.4). Phages were used to infect the amber-codon-suppressing TG1 *E. coli* cells for 30 min at 37 °C, which were later co-infected with 10^15^ M13K07 helper phages. Enriched phages were grown overnight at 37 °C in 2× Tryptone & Yeast extract medium supplemented with 100 µg/mL ampicillin and 70 µg/mL kanamycin. New produced phages were precipitated with ice-cold polyethylene glycol-6000/NaCl and resuspended in 1 mL PBS.

### Specificity by enzyme-linked immunosorbent assay (ELISA)

Nanobodies were expressed in TG1 *E. coli* by adding isopropyl β-d-1-thiogalactopyranoside (IPTG) to cultures at a final concentration of 1 mM and incubating overnight at 28 °C. Extraction of periplasmic proteins including hemagglutinin(HA)-tagged nanobodies was performed by osmotic shock with 4× Tris/EDTA/sucrose and ddH_2_O. ELISA was performed by adding bacterial periplasmic extracts to 96-well NUNC MaxiSorp plates (Thermo Scientific), previously coated with 2 µg/mL of ABCC3-derived peptide in 0.1 M NaHCO_3_ (5 µg/mL for measuring binding specificity). Absorbance at 405 nm was measured on a Synergy HT multi-mode microplate reader (BioTek), after incubation with mouse anti-HA (1:2000; Sigma-Aldrich) and goat anti-mouse IgG conjugated with alkaline phosphatase (1:2000; Sigma-Aldrich) antibodies, and alkaline phosphatase substrate (Sigma-Aldrich). Potential clones targeting ABCC3 rendered 1.5 higher signal than its negative control without antigen. Genetic sequences of specific nanobodies were amplified by colony PCR with primers RP (5'-TCACACAGGAAACAGCTATGAC-3') and GIII (5'-CCACAGACAGCCCTCATAG-3'), and sequenced at Macrogen (The Netherlands).

### Production of nanobodies

Nanobodies with complete sequences were recloned from pHEN4^12^ into the pHEN6^64^ expression vector, to replace their HA-tag by a hexahistidine(His)-tag for further purification. Briefly, similar colony PCR with primers A6E (5'-GATGTGCAGCTGCAGGAGTCTGGAGGAGG-3') and 38 (5'-GGACTAGTG CGGCCGCTGGAGACGGTGACCTGGGT-3') yielded products of 400 bp, further cloned into pHEN6 with *Pst*I and *BstE*II (*Eco91*I) (Thermo Scientific). Conservation of sequences was verified by PCR with FP (5'-CGCCAGGGTTTTCCCAGTCACGAC-3') and RP (5'-TCACACAGGAAACAGCTATGAC-3'), with further sequencing. Expression of nanobodies encoded in the pHEN6 vector was performed in WK6 *E. coli* as previously described^64^. Briefly, 1 mM of IPTG was added to cultures to induce nanobody expression overnight at 28 ˚C. Periplasmic extracts were obtained by osmotic shock and dialyzed on Spectra/Por dialysis membrane tubing of MWCO 3.5 kDa (Spectrum laboratories, Inc) in PBS. His-tagged nanobodies were purified using an increasing gradient of 0.5 M imidazole on immobilized metal affinity chromatography columns in an ÄKTA protein purification system (GE Healthcare). Nanobody concentration was measured on a Synergy HT multi-mode microplate reader (BioTek) after incubation with Bradford reagent (Bio-Rad Laboratories, Inc).

### Flow cytometry

To analyze the expression levels of ABCC3, 4 × 10^5^ cells were counted using a Neubauer chamber using Trypan Blue Solution 0.4% (Fisher Scientific). After incubation in blocking and permeabilizing buffer (PBS/saponin 0.1%/FBS 5%) for 15 min at 4 °C, cells were stained with anti-ABCC3 monoclonal antibody (mAb, 1:150; clone M3II-9; Fisher Scientific) and APC goat anti-mouse IgG antibody (1:1500; clone Poly4053; Biolegend) for 20 min at 4 °C. To stain with nanobodies targeting ABCC3, cells were detached with 10 mM EDTA (Fisher Scientific). Upon incubation in blocking buffer (PBS/1% BSA) for 15 min at 4 °C, cells were stained with 5 µg of anti-ABCC3 nanobody followed by ReadyTag anti-6-His mAb (1:1500; clone 6-HIS, BioXCell) and APC goat anti-mouse IgG antibody (1:1500; clone Poly4053, Biolegend) for 20 min at 4 °C. Cells were analyzed on a Gallios flow cytometer (Beckman Coulter) and results were visualized at Kaluza analysis software (Beckman Coulter). To estimate the equilibrium dissociation constant (K_D_), nanobodies were conjugated to fluorescein-5-isothiocyanate using FluoroTag™ FITC Conjugation Kit (Sigma-Aldrich), and total and non-specific binding was analyzed with GraphPad Prism v.6.01.

### Animal ethics statement and experimental design

Athymic Nude-Foxn1^nu/nu^ mice were purchased from Envigo (Spain). Mice were housed in animal experimental core facilities of the Aragon Institute of Health Sciences (IACS) at the Biomedical Research Center of Aragon (CIBA, Zaragoza, Spain). All animal experiments were approved and carried out according to the Ethical Committee for Animal Experimentation of the University of Zaragoza, under the protocol project No. PI60/20. Mice were cared for in accordance with the European 2010/63/UE and Spanish national RD1386/2018 legal statements.

Subcutaneous xenograft models were established (1 × 10^6^ cells; 6–8 weeks athymic nude mice). Nanobodies were administered intraperitoneally (i.p.) at 15 mg·kg^−1^ in mice bearing tumors (1000 mm^3^). Mice were sacrificed 1 h after administration; tumors were mechanically disaggregated and incubated in 10 mM EDTA (Fisher Scientific). The targeting profile of nanobodies was evaluated by prospective ex vivo flow cytometry in collected tissues (liver, kidney, brain, blood, and tumors).

For orthotopic xenografts, U-87 MG and 251 cells were transduced with a TK-GFP-Luciferase reporter plasmid (TGL) to monitor tumor growth by bioluminescence measurements. To develop intracranial tumors, 6–8 weeks athymic nude mice were anesthetized by inhalation of 3% isoflurane and were subcutaneously injected with 50 μL of the local anesthetic 0.25% bupivacaine at the surgical site. Mice were intracranially injected with 2 μL containing 5·10^5^ U-87 MG-TGL or 251-MG-TGL cells resuspended in PBS, using a fixed stereotactic apparatus (Stoelting). Injections were made to the right frontal cortex, approximately 1 mm caudal and 1.5 mm lateral from bregma, and at a depth of 2 mm using a Hamilton syringe (Hamilton) as described previously^65,66^. Tumor growth was monitored by bioluminescence. Mice were injected with D-Luciferin (150 mg/kg). 15 min after injection, images were acquired for 5 s using an IVIS Lumina XRMS equipment (Perkin Elmer). Bioluminescence analysis was performed using Living Image software, version 2.50. Nanobodies were administered as described above in mice with tumors in a positive growth phase determined by bioluminescence (10^6^ to 10^8^ p/s/cm^2^/sr of average radiance). Mice were sacrificed 1 h after administration, and tumors were processed as described above.

To evaluate the BBB disruption, 6–8 weeks athymic nude littermates bearing similar intracranial tumors were injected i.p. with 400 μL of 4% Evan's blue dye (Sigma). One hour after injection, mice were sacrificed by CO_2_ inhalation and perfused with acidified fixative (1% PFA in 0.05 mM citrate buffer, pH 3.5). Collected brain tumors and samples of their contralateral hemisphere were incubated in 700 μL of formamide (Sigma) to extract Evan's blue at 60 °C overnight. Absorbance was measured at 610 nm and 740 nm on a Synergy HT multi-mode microplate reader (BioTek). Non-injected or intracranially injected with 2 μL of PBS (mock surgery), 6–8 weeks athymic nude littermates were included as intact brain controls.

To evaluate the bioavailability of nanobodies plasma and urine samples were obtained from mice sacrificed 15 min, 30 min, 1 h, 4 h, and 24 h after administration of nanobodies. Protein content was measured in plasma and urine samples using a Synergy HT multi-mode microplate reader (BioTek) after incubation with Bradford reagent (Bio-Rad Laboratories, Inc). A volume containing 5 μg of protein for plasma and 1 μg for urine was used for immunoblot detection. ReadyTag anti-6-His mAb (1:1,000; clone 6-HIS, BioXCell) and goat anti-mouse IgG IRDye800CW antibody (1:20,000; LI-COR) allowed detection of His-tagged nanobodies in an Odyssey CLx Imaging System (LI-COR). Vehicle-treated healthy mice were used as controls.

### Experimental statement

All experiments and methods were performed in accordance with relevant guidelines and regulations.

## Supplementary Information

Supplementary Information.

## Data Availability

Data supporting the findings of this manuscript are available from the corresponding author upon reasonable request. Data for bioinformatic analysis are deposited in publicly available patient datasets (e.g. RNA-Seq data from The Cancer Genome Atlas (TCGA; https://www.cancer.gov/tcga); gene expression and patient survival data from TCGA Project (TCGA-GBM and TCGA-GBMLGG), the Chinese Glioma Genome Atlas (CGGA, http://www.cgga.org.cn), and the Repository for Molecular Brain Neoplasia Data (REMBRANDT, https://wiki.cancerimagingarchive.net/display/Public/REMBRANDT).
