# Supplementary material for: Nanobodies targeting ABCC3 for immunotargeted applications in glioblastoma
Source: Sci Rep. 2022 Dec 30;12:22581. doi: 10.1038/s41598-022-27161-3 (PMC9803684; doi:10.1038/s41598-022-27161-3)
Supplement: Supplementary file 1 — Supplementary Information. [file 41598_2022_27161_MOESM1_ESM.docx]

Nanobodies Targeting ABCC3 for Immunotargeted Applications in Glioblastoma

Eduardo Ruiz-López^1^, Ivana Jovčevska^2^, Ruth González-Gómez^1^, Héctor Tejero^3^, Fátima Al-Shahrour^3^, Serge Muyldermans^4^ and Alberto J. Schuhmacher^1,5,^*

^1^ Molecular Oncology Group, Instituto de Investigación Sanitaria Aragón (IIS Aragón), 50009 Zaragoza, Spain; [eruiz@iisaragon.es](mailto:eruiz@iisaragon.es) (E.R.-L.); [rgongalez@iisaragon.es](mailto:rgongalez@iisaragon.es) (R.G.-G.)

^2^ Center for Functional Genomics and Biochips, Institute of Biochemistry and Molecular Genetics, Faculty of Medicine, University of Ljubljana, Ljubljana, Slovenia; ivana.jovcevska@mf.uni-lj.si (I.J.)

^3^ Bioinformatics Unit, Centro Nacional de Investigaciones Oncológicas (CNIO), 28029 Madrid, Spain; [falshahrour@cnio.es](mailto:falshahrour@cnio.es) (F.A-S.), htejero@cnio.es (H.T.)

^4^ Cellular and Molecular Immunology, Vrije Universiteit Brussel, Pleinlaan 2, 1050, Brussels, Belgium. [serge.muyldermans@vub.be](mailto:serge.muyldermans@vub.be) (S.M.)

^5^ Fundación Aragonesa para la Investigación y el Desarrollo (ARAID), 50018 Zaragoza, Spain; [ajimenez@iisaragon.es](mailto:ajimenez@iisaragon.es) (A.J.S.)

***** Correspondence: [ajimenez@iisaragon.es](mailto:ajimenez@iisaragon.es) (A.J.S.)

**Supplementary Information**

**
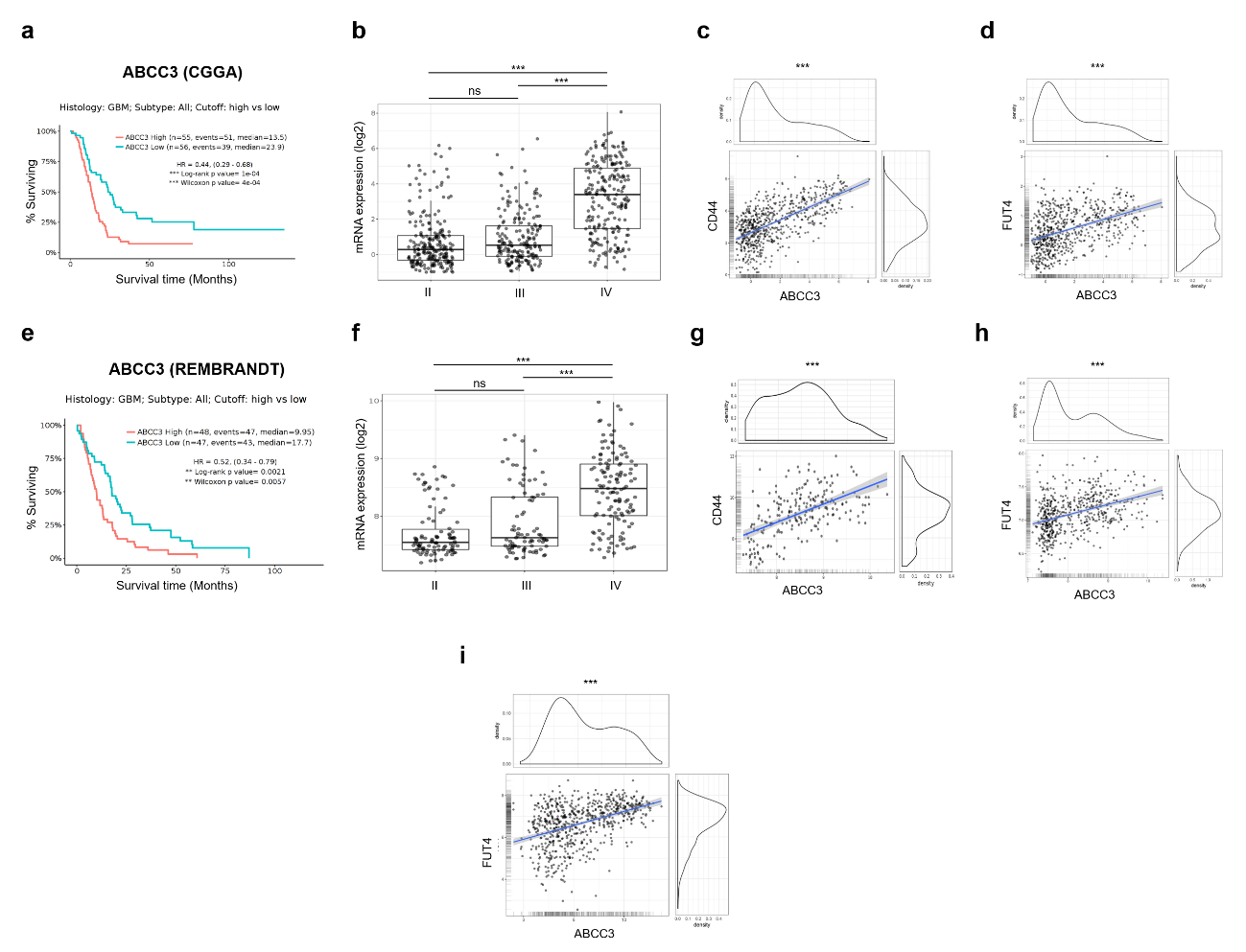
**

**Supplementary Figure S1. Analysis of the expression of *ABCC3* in multiple datasets**. (**a-d**) Chinese Glioma Genome Atlas (CGGA) dataset available from http://www.cgga.org.cn/ (RNA-Seq, GlioVis) [57]. (**a**) Kaplan-Meier curves for overall survival for *ABCC3*. (**b**) *ABCC3* expression increases with tumor grade (mean±SEM). Grade II (n=232), Grade III (n=194), Grade IV (n=223). *ABCC3* correlates with *CD44* (**c**) and *FUT4* (**d**) expression. (**e-h**) Repository of Molecular Brain Neoplasia Data (REMBRANDT) dataset available from https://wiki.cancerimagingarchive.net/display/Public/REMBRANDT (RNA-Seq, GlioVis) [58]. (**e**) Kaplan-Meier curves for overall survival for *ABCC3*. (**f**) *ABCC3* expression increases with tumor grade (mean±SEM). Grade II (n=98), Grade III (n=85), Grade IV (n=130). *ABCC3* correlates with *CD44* (**g**) and *FUT4* (**h**) expression. (**i**) The Cancer Genome Atlas (TGGA) dataset available from <https://www.cancer.gov/tcga> (RNA-Seq, GlioVis). *ABCC3* expression correlates with *FUT4*. Log-rank and Wilcoxon test for (a) and (e), ***p<0.001; HSD test for (b) and (f), ***p<0.001, ns; Pearson's product-moment correlation for (c), (d) , (g), (h) and (i). ***p<0.001.

**
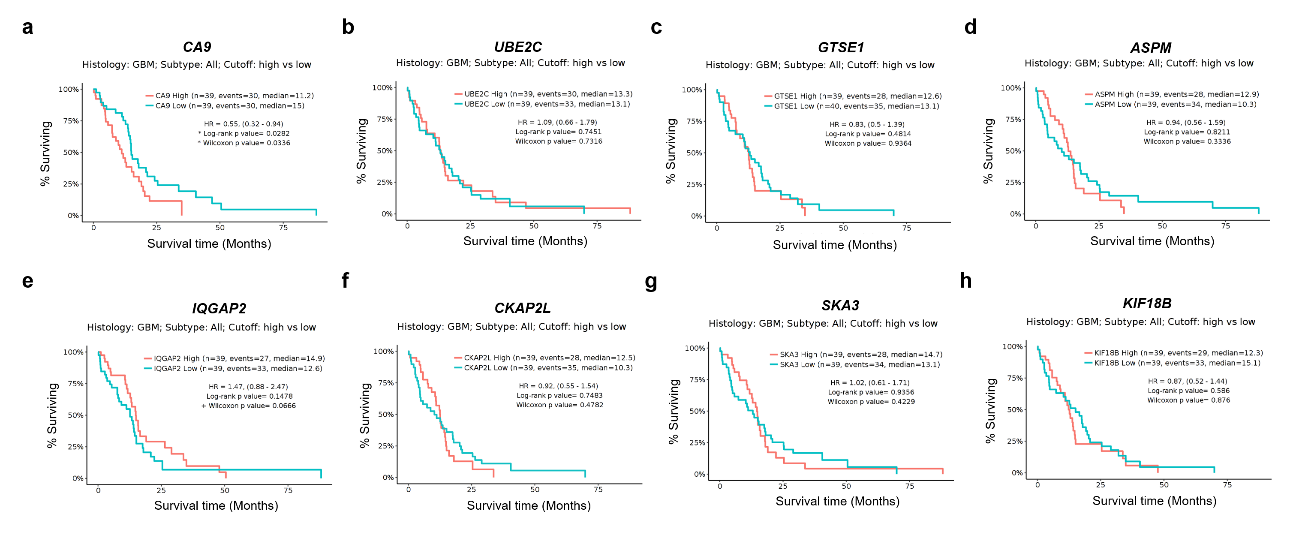
**

**Supplementary Figure S2. Overall survival of the most differentially expressed membrane-associated genes in glioblastoma vs. healthy tissue (Log_2_FC>4).** Kaplan-Meier curves for overall survival in the TCGA-GBM dataset (RNA-Seq, GlioVis) for other differentially expressed genes in glioblastoma vs. normal tissue (Log_2_FC>4)(**a**) *CA9*, (**b**) *UBE2C*, (**c**) *GSTE1*, (**d**) *ASPM*, (**e**) *IQGAP2*, (**f**) *CKAP2L*, (**g**) *SKA3*, and (**h**) *KIF18B*. Log-rank and Wilcoxon test for survival curves. ns.

**
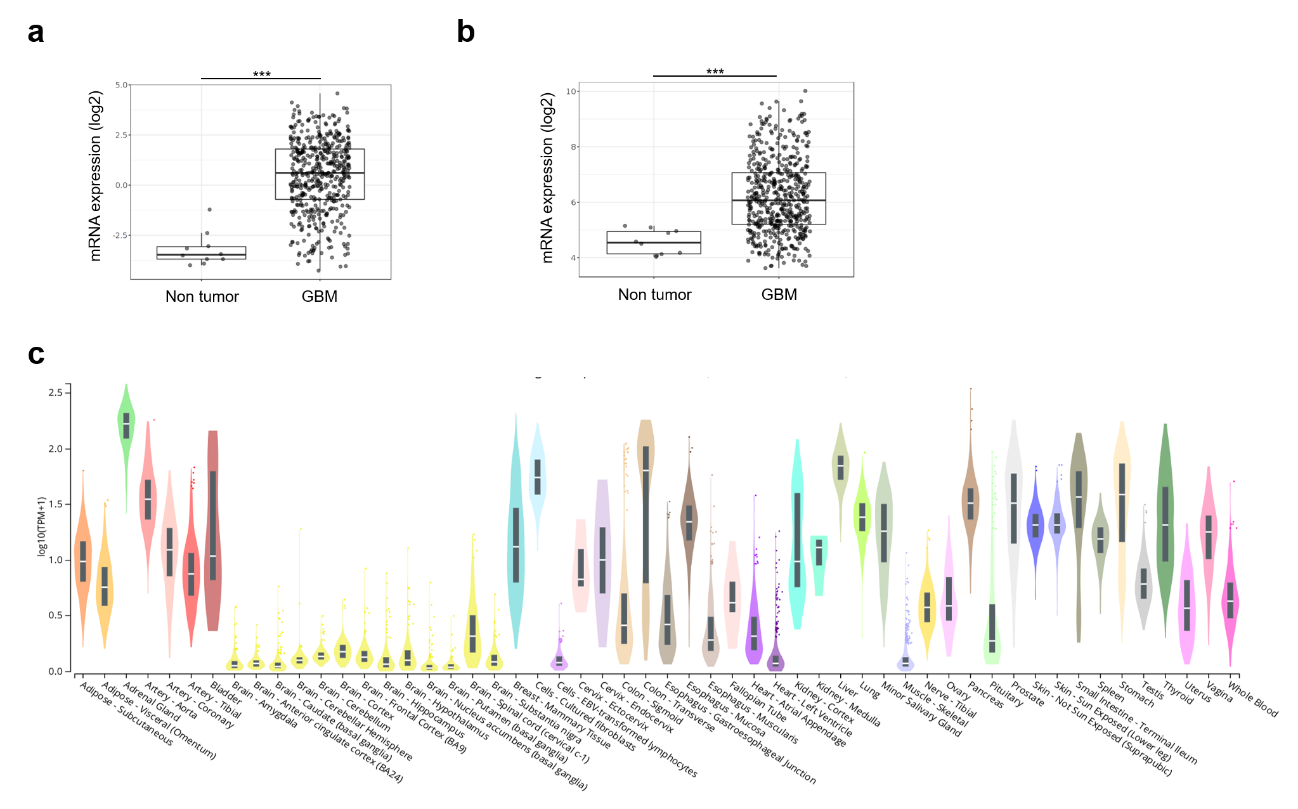
**

**Supplementary Figure S3**. **Brain tissues present low levels of *ABCC3* expression.** *ABCC3* expression analysis in glioblastoma and control brain samples in the ATCC-GBM dataset (GlioVis). (**a**) RNA expression determined by Agilent-4502A Custom Gene Expression Microarray (mean±SEM). Glioblastoma (n=489) and control brain (n=10) samples. (**b**) RNA expression determined by The GeneChip Human Genome U133A Array (HG-U133A; mean±SEM). Glioblastoma (n=528) and control brain (n=10) samples. HSD test for (a) and (b). ***p<0.001. (**c**) Bulk tissue gene expression for *ABCC3* in normal tissues. GTEx Analysis Release V8 (dbGaP Accession phs000424.v8.p2). Expression values are shown in TPM (Transcripts Per Million), calculated from a gene model with isoforms collapsed to a single gene. No other normalization steps have been applied. Box plots are shown as median and 25th and 75th percentiles; points are displayed as outliers if they are above or below 1.5 times the interquartile range. (ENSG00000108846.15).

**
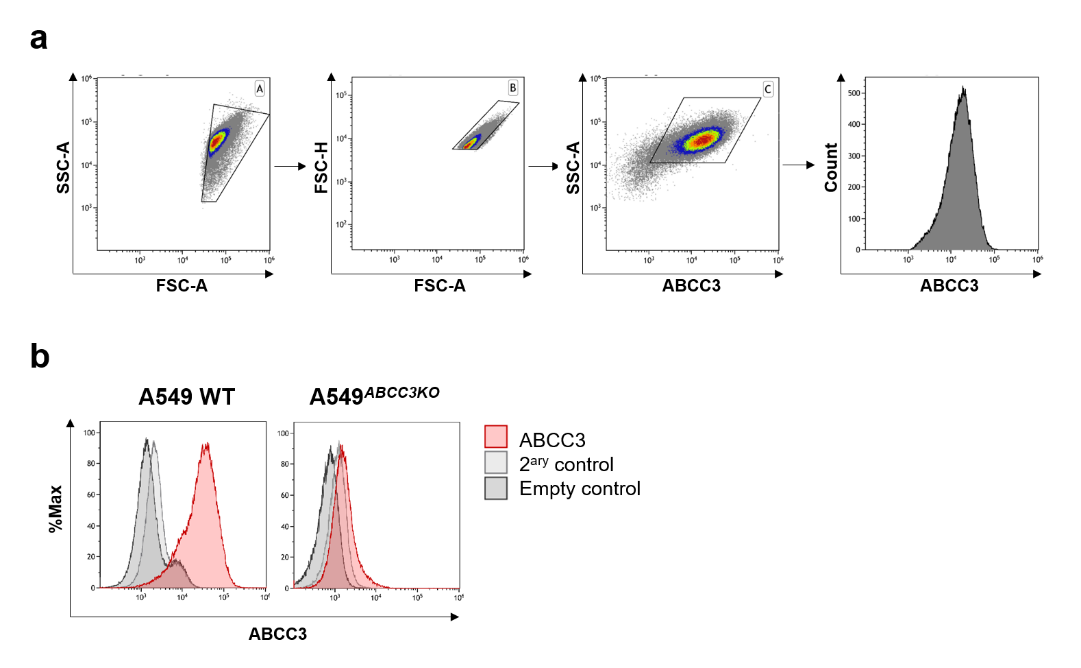
**

**Supplementary Figure S4.** **Analysis of ABCC3 by flow cytometry.** (**a**) Representative flow cytometry gating strategy for analyzing ABCC3 expression in different adherent cell lines and tissues with antibodies and/or nanobodies. (**b**) Evaluation of the ABCC3 expression in A549 WT and A549*^ABCC3KO^* control cell lines by flow cytometry with commercial mAb (clone M3II-9).

**
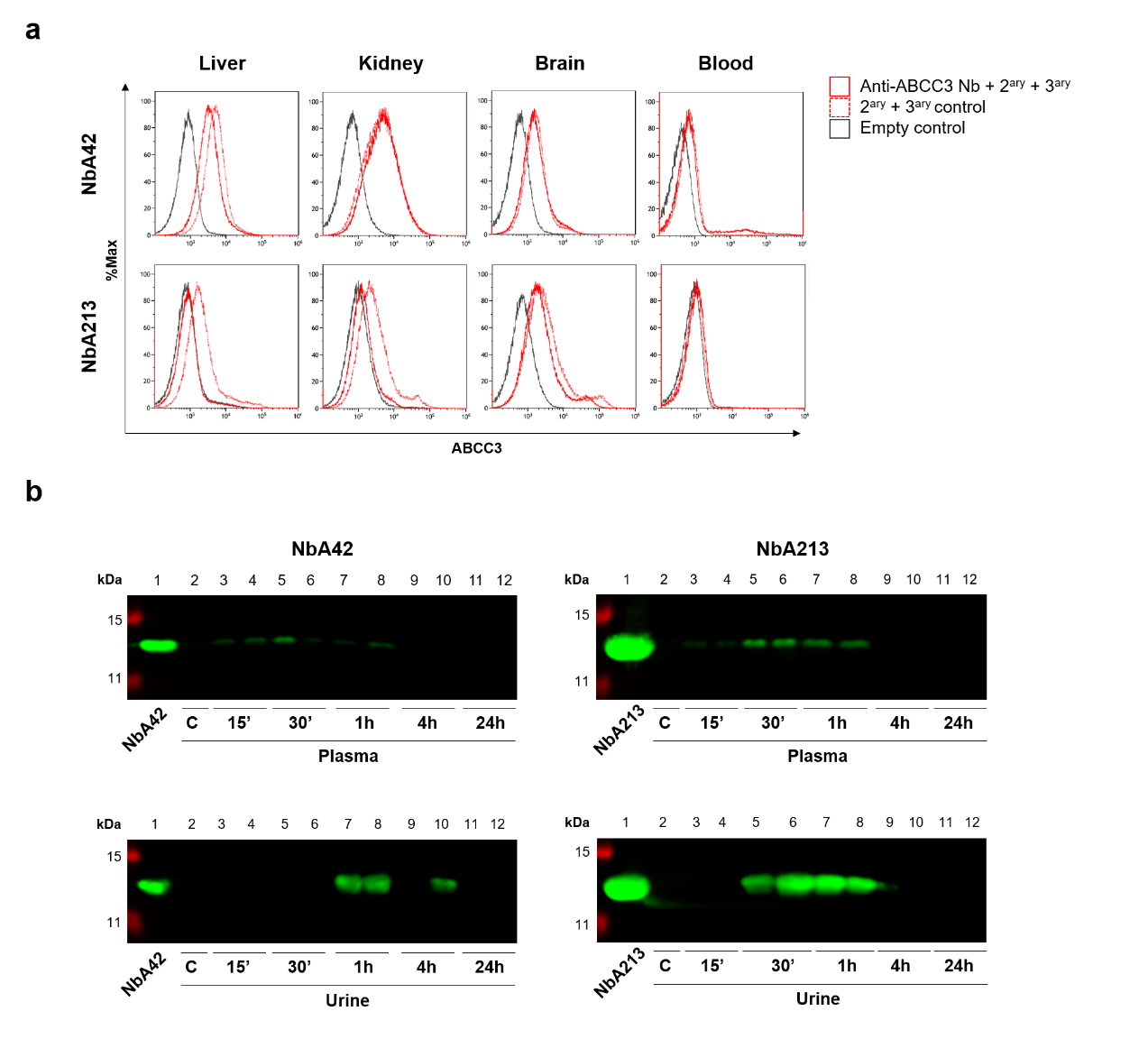
**

**Supplementary Figure S5. Pharmacokinetic profile of anti-ABCC3 nanobodies after systemic injection in mice**. (**a, b**) Tissue analysis of the biodistribution and clearance of NbA42 and NbA213. Both nanobodies were administered intraperitoneally (i.p.) at 15 mg·kg^-1^. (**a**) Off-target detection was examined using prospective *ex vivo* flow cytometry studies of the extracted organs (liver, kidney, brain, and blood), 1 h after administration. (**b**) Detection of the His-tagged NbA42 and NbA213 in blood plasma and urine, at different times (15 min, 30 min, 1 h, 4 h and 24 h) after systemic i.p. administration. Lane 1: control of the purified nanobody (0.5 μg). Lane 2: blood plasma/urine from a vehicle treated mouse. Lanes 3-12: blood plasma/urine from mouse with i.p. administration of nanobodies after 15 min (lanes 3, 4), 30 min (lanes 5, 6), 1 h (lanes 7, 8), 4 h (lanes 9, 10) and 24 h (lanes 11, 12). Analysis was performed by western blot using a ReadyTag anti-6-His mAb (1:1,000; clone 6-HIS, BioXCell) and goat anti-mouse IgG IRDye800CW antibody (1:20,000; LI-COR). PageRuler Prestained NIR Protein Ladder (Thermo Scientific) was utilized as standard of MW of proteins. Original western blots are presented in Supplementary Figure 7.


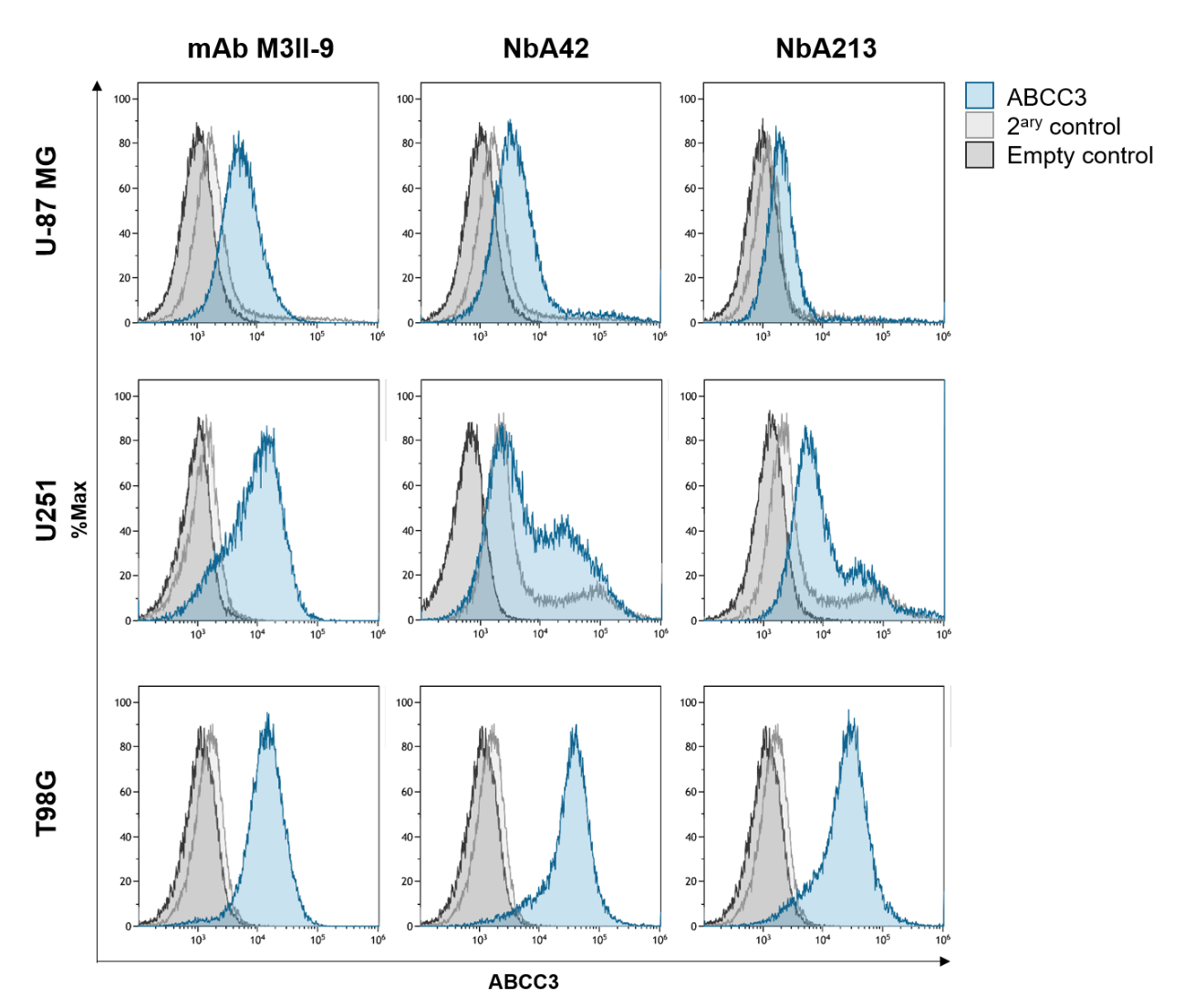


**Supplementary Figure S6. Analysis of ABCC3 in glioblastoma cell lines by flow cytometry.** Evaluation in U-87 MG, U251 and T98G cell lines with control commercial mAb (clone M3II-9) and anti-ABCC3 nanobodies (NbA42 and NbA213).


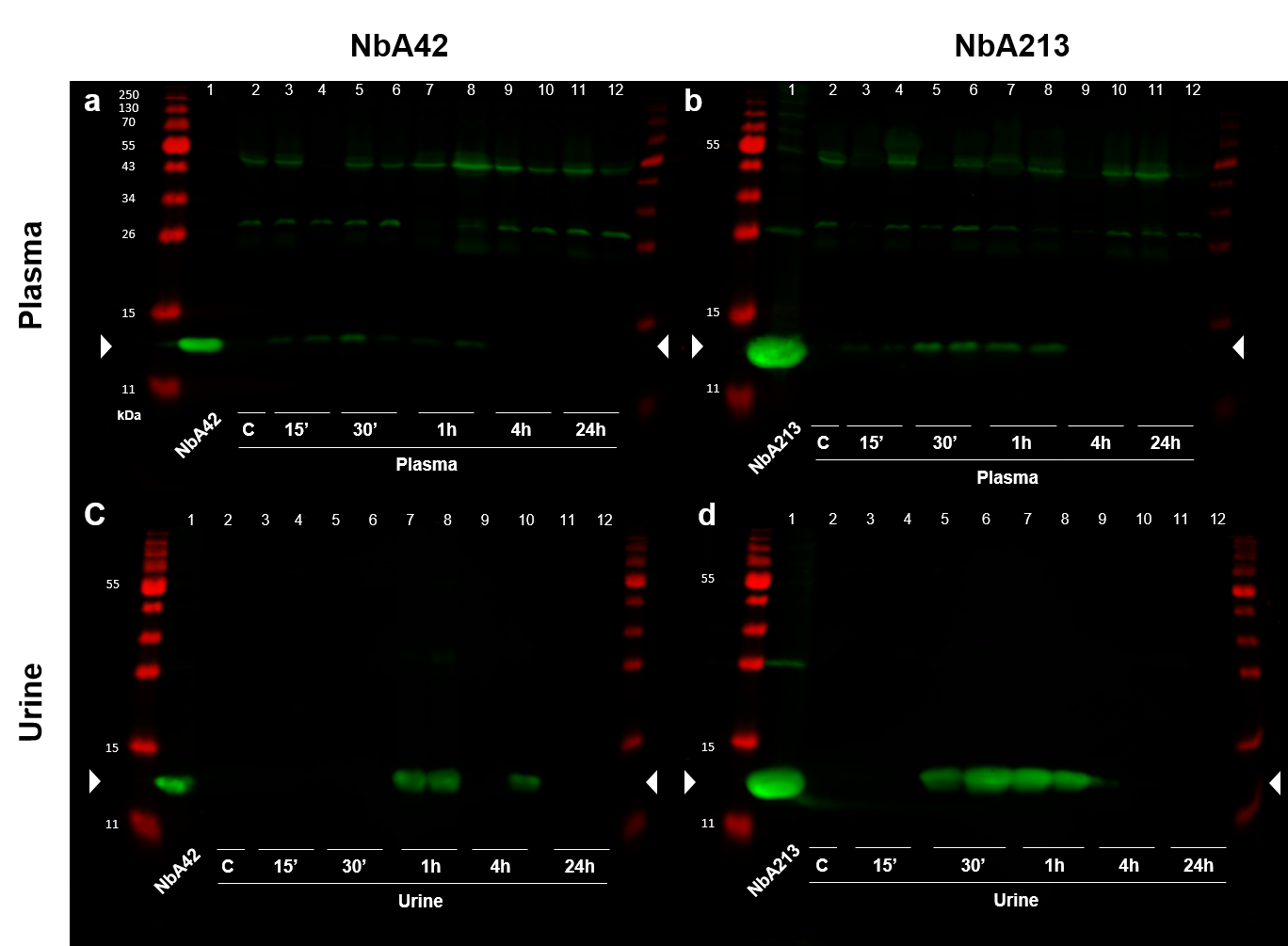


**Supplementary Figure S7. Original immuno blots for detection of the His-tagged NbA42 and NbA213 in blood plasma and urine.** Detection of NbA42 (**a**) and NbA213 (**b**) in plasma, and detection of NbA42 (**c**) and NbA213 (**d**) in urine, at different times (15 min, 30 min, 1 h, 4 h and 24 h) after systemic i.p. administration. For all the blots: Lane 1: control purified nanobody (0.5 µg). Lane 2: blood plasma/urine from a vehicle treated mouse. Lanes 3-12: blood plasma/urine from mouse with i.p. administration of nanobodies after 15 min (3, 4), 30 min (5, 6), 1 h (7, 8), 4 h (9, 10) and 24 h (11, 12). ReadyTag anti-6-His mAb (1:1,000; clone 6-HIS, BioXCell) and goat anti-mouse IgG IRDye800CW antibody (1:20,000; LI-COR) were used for visualization. White arrows indicate the molecular weight (MW) of bands corresponding to detected nanobodies. PageRuler Prestained NIR Protein Ladder (Thermo Scientific) was utilized as standard of MW of proteins.

| **No.** | **Gene** | **LogFC** | **AveExpr** | **t** | **p** | **Adj. P** | **B** | **Ensembl** | **Gene Ontology** | **Location** | **Evidence** |
| --- | --- | --- | --- | --- | --- | --- | --- | --- | --- | --- | --- |
| 1 | ***UBE2C*** | 8,2906 | 4,6992 | 8,1157 | *** | *** | 20,219 | ENSP00000348838 | GO:0005886 | Plasma membrane | + |
| 2 | ***ABCC3*** | 5,3034 | 5,0883 | 3,0139 | ** | ** | -2,122 | ENSP00000285238 | GO:0005886 | Plasma membrane | ++ |
| 3 | ***GTSE1*** | 5,2905 | 3,5219 | 5,7849 | *** | *** | 8,342 | ENSP00000415430 | GO:0005886 | Plasma membrane | ++ |
| 4 | ***CA9*** | 5,1798 | 2,9629 | 2,7261 | ** | * | -2,854 | ENSP00000367608 | GO:0005886 | Plasma membrane | ++ |
| 5 | ***ASPM*** | 4,9348 | 3,5202 | 4,8999 | *** | *** | 4,487 | ENSP00000356379 | GO:0005886 | Plasma membrane | +++ |
| 6 | ***IQGAP2*** | 4,7854 | 4,4876 | 5,4021 | *** | *** | 6,607 | ENSP00000274364 | GO:0005886 | Plasma membrane | ++ |
| 7 | ***CKAP2L*** | 4,3385 | 3,0900 | 5,5537 | *** | *** | 7,294 | ENSP00000305204 | GO:0005886 | Plasma membrane | ++ |
| 8 | ***SKA3*** | 4,2011 | 3,1054 | 5,0547 | *** | *** | 5,126 | ENSP00000319417 | GO:0005886 | Plasma membrane | +++ |
| 9 | ***KIF18B*** | 4,0671 | 3,6962 | 3,9447 | *** | *** | 0,818 | ENSP00000341466 | GO:0005886 | Plasma membrane | + |
| 10 | ***TNFRSF12A*** | 3,9796 | 5,8899 | 4,5476 | *** | *** | 2,883 | ENSP00000326737 | GO:0005886 | Plasma membrane | + |
| 11 | ***ANXA1*** | 3,8479 | 7,8900 | 5,8338 | *** | *** | 8,194 | ENSP00000257497 | GO:0005886 | Plasma membrane | +++ |
| 12 | ***EGFR*** | 3,8165 | 7,5114 | 2,9028 | ** | * | -2,962 | ENSP00000275493 | GO:0005886 | Plasma membrane | +++ |
| 13 | ***ERCC6L*** | 3,6865 | 1,4201 | 7,0301 | *** | *** | 14,413 | ENSP00000334675 | GO:0005886 | Plasma membrane | + |
| 14 | ***IFI30*** | 3,6186 | 6,8882 | 5,4204 | *** | *** | 6,393 | ENSP00000384886 | GO:0005886 | Plasma membrane | + |
| 15 | ***C11orf82*** | 3,6175 | 2,9258 | 6,0186 | *** | *** | 9,428 | ENSP00000414687 | GO:0005886 | Plasma membrane | ++ |
| 16 | ***PLAU*** | 3,5981 | 5,0348 | 3,2832 | ** | ** | -1,483 | ENSP00000361850 | GO:0005886 | Plasma membrane | ++ |
| 17 | ***NMB*** | 3,4016 | 6,0543 | 3,9228 | *** | *** | 0,438 | ENSP00000378089 | GO:0005886 | Plasma membrane | + |
| 18 | ***CELSR1*** | 3,3697 | 2,8754 | 2,6127 | ** | * | -3,152 | ENSP00000262738 | GO:0005886 | Plasma membrane | ++ |
| 19 | ***CD44*** | 3,3631 | 8,9725 | 6,9380 | *** | *** | 13,612 | ENSP00000398632 | GO:0005886 | Plasma membrane | +++ |
| 20 | ***VSIG4*** | 3,1986 | 6,9156 | 4,4443 | *** | *** | 2,201 | ENSP00000363869 | GO:0005886 | Plasma membrane | + |
| 21 | ***CASP4*** | 3,1812 | 4,3934 | 4,3983 | *** | *** | 2,383 | ENSP00000388566 | GO:0005886 | Plasma membrane | ++ |
| 22 | ***S100A12*** | 3,1466 | -1,9932 | 4,3407 | *** | *** | 2,158 | ENSP00000357726 | GO:0005886 | Plasma membrane | + |
| 23 | ***S100A2*** | 3,1413 | 1,1318 | 5,1981 | *** | *** | 5,735 | ENSP00000357697 | GO:0005886 | Plasma membrane | + |
| 24 | ***FANCI*** | 3,0937 | 5,1238 | 4,8447 | *** | *** | 4,080 | ENSP00000310842 | GO:0005886 | Plasma membrane | +++ |
| 25 | ***PLEKHA4*** | 3,0905 | 5,8612 | 4,3049 | *** | *** | 1,821 | ENSP00000263265 | GO:0005886 | Plasma membrane | ++ |
| 26 | ***PYGL*** | 3,0836 | 6,0545 | 5,7428 | *** | *** | 7,926 | ENSP00000216392 | GO:0005886 | Plasma membrane | +++ |
| 27 | ***AFAP1L1*** | 2,9092 | 4,5029 | 3,8996 | *** | *** | 0,514 | ENSP00000296721 | GO:0005886 | Plasma membrane | + |
| 28 | ***SMO*** | 2,8188 | 5,9231 | 5,8959 | *** | *** | 8,628 | ENSP00000249373 | GO:0005886 | Plasma membrane | ++ |
| 29 | ***FAM129A*** | 2,7757 | 5,2049 | 3,7669 | *** | *** | -0,061 | ENSP00000356481 | GO:0005886 | Plasma membrane | +++ |
| 30 | ***DLEU1*** | 2,7196 | 2,7726 | 5,7233 | *** | *** | 8,049 | ENSP00000367422 | GO:0005886 | Plasma membrane | ++ |
| 31 | ***ADAM12*** | 2,7142 | 4,6222 | 2,7375 | ** | * | -3,079 | ENSP00000357668 | GO:0005886 | Plasma membrane | +++ |
| 32 | ***LIMA1*** | 2,6941 | 7,1689 | 6,8902 | *** | *** | 13,423 | ENSP00000378400 | GO:0005886 | Plasma membrane | ++ |
| 33 | ***LRRC17*** | 2,5356 | 4,0277 | 2,7015 | ** | * | -3,113 | ENSP00000344242 | GO:0005886 | Plasma membrane | ++ |
| 34 | ***B2M*** | 2,5170 | 11,1911 | 7,5807 | *** | *** | 17,102 | ENSP00000452780 | GO:0005886 | Plasma membrane | ++ |
| 35 | ***HSPG2*** | 2,4861 | 6,6201 | 4,4698 | *** | *** | 2,240 | ENSP00000363827 | GO:0005886 | Plasma membrane | + |
| 36 | ***SPATA12*** | 2,4724 | -1,7812 | 5,6123 | *** | *** | 7,520 | ENSP00000335392 | GO:0005886 | Plasma membrane | + |
| 37 | ***COL6A2*** | 2,4399 | 7,1311 | 2,8534 | ** | * | -3,240 | ENSP00000300527 | GO:0005886 | Plasma membrane | ++ |
| 38 | ***ABCB4*** | 2,4006 | 1,6807 | 4,6872 | *** | *** | 3,617 | ENSP00000265723 | GO:0005886 | Plasma membrane | + |
| 39 | ***JAG1*** | 2,3600 | 6,6900 | 5,1948 | *** | *** | 5,242 | ENSP00000254958 | GO:0005886 | Plasma membrane | ++ |
| 40 | ***MUC1*** | 2,3428 | 3,3364 | 3,5377 | *** | ** | -0,635 | ENSP00000357380 | GO:0005886 | Plasma membrane | + |
| 41 | ***PDIA5*** | 2,3374 | 3,4369 | 4,2857 | *** | *** | 1,968 | ENSP00000323313 | GO:0005886 | Plasma membrane | + |
| 42 | ***HMOX1*** | 2,2861 | 6,9921 | 4,1559 | *** | *** | 0,969 | ENSP00000216117 | GO:0005886 | Plasma membrane | +++ |
| 43 | ***LGALS3*** | 2,2767 | 7,0614 | 3,1322 | ** | ** | -2,461 | ENSP00000254301 | GO:0005886 | Plasma membrane | ++ |
| 44 | ***FLNA*** | 2,2692 | 9,2951 | 5,9707 | *** | *** | 8,742 | ENSP00000358866 | GO:0005886 | Plasma membrane | ++ |
| 45 | ***C17orf53*** | 2,2677 | 1,9703 | 5,4728 | *** | *** | 6,924 | ENSP00000313500 | GO:0005886 | Plasma membrane | + |
| 46 | ***STK17B*** | 2,2655 | 4,9651 | 5,1866 | *** | *** | 5,456 | ENSP00000263955 | GO:0005886 | Plasma membrane | +++ |
| 47 | ***S100A16*** | 2,2591 | 7,7183 | 4,9854 | *** | *** | 4,248 | ENSP00000357692 | GO:0005886 | Plasma membrane | ++ |
| 48 | ***NEDD1*** | 2,2562 | 4,5604 | 7,1588 | *** | *** | 15,065 | ENSP00000451211 | GO:0005886 | Plasma membrane | + |
| 49 | ***C5AR1*** | 2,2494 | 4,3821 | 2,7385 | ** | * | -3,105 | ENSP00000347197 | GO:0005886 | Plasma membrane | ++ |
| 50 | ***HTRA3*** | 2,2099 | 2,7271 | 2,9357 | ** | * | -2,380 | ENSP00000303766 | GO:0005886 | Plasma membrane | ++ |
| 51 | ***HAPLN3*** | 2,2063 | 2,8787 | 4,4428 | *** | *** | 2,601 | ENSP00000352606 | GO:0005886 | Plasma membrane | ++ |
| 52 | ***TMEM218*** | 2,1915 | 4,8583 | 5,9884 | *** | *** | 9,131 | ENSP00000279968 | GO:0005886 | Plasma membrane | ++ |
| 53 | ***TES*** | 2,1675 | 3,0752 | 4,3850 | *** | *** | 2,362 | ENSP00000350937 | GO:0005886 | Plasma membrane | + |
| 54 | ***DIAPH3*** | 2,1557 | 2,3229 | 4,1834 | *** | *** | 1,653 | ENSP00000383178 | GO:0005886 | Plasma membrane | ++ |
| 55 | ***LAMA4*** | 2,1553 | 6,2405 | 4,5521 | *** | *** | 2,578 | ENSP00000230538 | GO:0005886 | Plasma membrane | ++ |
| 56 | ***RAB13*** | 2,1393 | 6,1959 | 6,6455 | *** | *** | 12,207 | ENSP00000357564 | GO:0005886 | Plasma membrane | ++ |
| 57 | ***MSN*** | 2,1377 | 9,0150 | 6,9878 | *** | *** | 13,877 | ENSP00000353408 | GO:0005886 | Plasma membrane | +++ |
| 58 | ***SLC1A5*** | 2,1357 | 4,8710 | 3,8676 | *** | *** | 0,238 | ENSP00000444408 | GO:0005886 | Plasma membrane | ++ |
| 59 | ***CALD1*** | 2,1313 | 7,9997 | 7,6648 | *** | *** | 17,515 | ENSP00000354826 | GO:0005886 | Plasma membrane | ++ |
| 60 | ***KNTC1*** | 2,0828 | 4,0914 | 4,0988 | *** | *** | 1,174 | ENSP00000328236 | GO:0005886 | Plasma membrane | ++ |
| 61 | ***TRIP6*** | 2,0536 | 6,4916 | 5,2225 | *** | *** | 5,350 | ENSP00000200457 | GO:0005886 | Plasma membrane | ++ |
| 62 | ***CCDC15*** | 2,0533 | 1,7071 | 4,6411 | *** | *** | 3,423 | ENSP00000341684 | GO:0005886 | Plasma membrane | + |
| 63 | ***CDCA5*** | 2,0443 | 4,5317 | 3,4236 | *** | ** | -1,190 | ENSP00000275517 | GO:0005886 | Plasma membrane | + |
| 64 | ***PRC1*** | 2,0163 | 5,5735 | 4,1501 | *** | *** | 1,118 | ENSP00000377793 | GO:0005886 | Plasma membrane | ++ |
| 65 | ***EDNRA*** | 1,9779 | 4,6378 | 3,3664 | *** | ** | -1,400 | ENSP00000315011 | GO:0005886 | Plasma membrane | + |
| 66 | ***LGALS1*** | 1,9735 | 8,3511 | 4,5723 | *** | *** | 2,526 | ENSP00000215909 | GO:0005886 | Plasma membrane | + |
| 67 | ***SMAGP*** | 1,9511 | 1,6186 | 3,0165 | ** | ** | -2,095 | ENSP00000369446 | GO:0005886 | Plasma membrane | ++ |
| 68 | ***ODC1*** | 1,9424 | 7,7253 | 6,6690 | *** | *** | 12,212 | ENSP00000234111 | GO:0005886 | Plasma membrane | + |
| 69 | ***PLTP*** | 1,9242 | 8,4283 | 4,3976 | *** | *** | 1,836 | ENSP00000361508 | GO:0005886 | Plasma membrane | ++ |
| 70 | ***PALLD*** | 1,9119 | 7,2842 | 6,7191 | *** | *** | 12,480 | ENSP00000425556 | GO:0005886 | Plasma membrane | ++ |
| 71 | ***PHLDA1*** | 1,9041 | 7,8115 | 4,8692 | *** | *** | 3,746 | ENSP00000266671 | GO:0005886 | Plasma membrane | ++ |
| 72 | ***ZIC4*** | 1,8954 | 2,1283 | 3,0340 | ** | ** | -2,082 | ENSP00000435509 | GO:0005886 | Plasma membrane | + |
| 73 | ***ADAMTSL4*** | 1,8894 | 3,1239 | 2,7247 | ** | * | -3,012 | ENSP00000271643 | GO:0005886 | Plasma membrane | ++ |
| 74 | ***MYOF*** | 1,8815 | 5,3473 | 3,5369 | *** | ** | -1,011 | ENSP00000352208 | GO:0005886 | Plasma membrane | ++ |
| 75 | ***PSTPIP2*** | 1,8665 | 1,6557 | 3,3091 | ** | ** | -1,253 | ENSP00000387261 | GO:0005886 | Plasma membrane | ++ |
| 76 | ***FLT4*** | 1,8658 | 3,6275 | 2,9286 | ** | * | -2,539 | ENSP00000261937 | GO:0005886 | Plasma membrane | + |
| 77 | ***IL15RA*** | 1,8647 | 1,5241 | 3,9780 | *** | *** | 0,943 | ENSP00000369312 | GO:0005886 | Plasma membrane | ++ |
| 78 | ***ARHGAP18*** | 1,8637 | 4,7025 | 3,7900 | *** | *** | -0,042 | ENSP00000275189 | GO:0005886 | Plasma membrane | ++ |
| 79 | ***DDX60L*** | 1,8444 | 4,0409 | 3,7974 | *** | *** | 0,083 | ENSP00000260184 | GO:0005886 | Plasma membrane | ++ |
| 80 | ***TEX9*** | 1,8415 | 1,8761 | 3,6138 | *** | ** | -0,312 | ENSP00000342169 | GO:0005886 | Plasma membrane | ++ |
| 81 | ***ZNF20*** | 1,8257 | 3,1837 | 6,0281 | *** | *** | 9,433 | ENSP00000335437 | GO:0005886 | Plasma membrane | ++ |
| 82 | ***NEDD9*** | 1,8006 | 5,7938 | 4,1719 | *** | *** | 1,123 | ENSP00000368759 | GO:0005886 | Plasma membrane | + |
| 83 | ***DAB2*** | 1,7875 | 5,3306 | 3,7968 | *** | *** | -0,145 | ENSP00000313391 | GO:0005886 | Plasma membrane | ++ |
| 84 | ***OAS3*** | 1,7868 | 5,9224 | 2,8385 | ** | * | -3,206 | ENSP00000228928 | GO:0005886 | Plasma membrane | + |
| 85 | ***FAS*** | 1,7743 | 4,0529 | 2,7101 | ** | * | -3,197 | ENSP00000347979 | GO:0005886 | Plasma membrane | ++ |
| 86 | ***NUAK2*** | 1,7728 | 2,8347 | 2,7953 | ** | * | -2,811 | ENSP00000356125 | GO:0005886 | Plasma membrane | + |
| 87 | ***PRKD1*** | 1,7387 | 4,9852 | 5,4360 | *** | *** | 6,481 | ENSP00000333568 | GO:0005886 | Plasma membrane | ++ |
| 88 | ***SLC16A3*** | 1,7194 | 5,6134 | 2,9804 | ** | ** | -2,774 | ENSP00000376150 | GO:0005886 | Plasma membrane | ++ |
| 89 | ***LAX1*** | 1,7181 | -2,4145 | 2,9881 | ** | ** | -2,260 | ENSP00000406970 | GO:0005886 | Plasma membrane | ++ |
| 90 | ***KIRREL*** | 1,7128 | 2,8700 | 2,3009 | * | * | -4,013 | ENSP00000352138 | GO:0005886 | Plasma membrane | + |
| 91 | ***VASP*** | 1,7120 | 5,6574 | 6,4550 | *** | *** | 11,254 | ENSP00000245932 | GO:0005886 | Plasma membrane | + |
| 92 | ***PDGFC*** | 1,7095 | 5,0829 | 3,5861 | *** | ** | -0,824 | ENSP00000422464 | GO:0005886 | Plasma membrane | ++ |
| 93 | ***BRCA1*** | 1,7071 | 4,0543 | 4,0620 | *** | *** | 0,992 | ENSP00000418960 | GO:0005886 | Plasma membrane | ++ |
| 94 | ***PPP4C*** | 1,6994 | 6,6259 | 8,0559 | *** | *** | 19,716 | ENSP00000279387 | GO:0005886 | Plasma membrane | ++ |
| 95 | ***HSPB1*** | 1,6854 | 8,0808 | 4,7821 | *** | *** | 3,382 | ENSP00000248553 | GO:0005886 | Plasma membrane | ++ |
| 96 | ***GPR157*** | 1,6825 | -2,3914 | 2,9668 | ** | ** | -2,316 | ENSP00000366628 | GO:0005886 | Plasma membrane | + |
| 97 | ***ITGB1*** | 1,6506 | 7,9455 | 5,9086 | *** | *** | 8,432 | ENSP00000303351 | GO:0005886 | Plasma membrane | ++ |
| 98 | ***BTN3A3*** | 1,6426 | 4,7489 | 3,7387 | *** | ** | -0,262 | ENSP00000244519 | GO:0005886 | Plasma membrane | ++ |
| 99 | ***FYB*** | 1,6371 | 4,6600 | 2,5250 | * | * | -3,795 | ENSP00000425845 | GO:0005886 | Plasma membrane | +++ |
| 100 | ***TXNIP*** | 1,6362 | 8,0305 | 5,2513 | *** | *** | 5,397 | ENSP00000358323 | GO:0005886 | Plasma membrane | +++ |
| 101 | ***SLC38A6*** | 1,6238 | 3,2633 | 4,4965 | *** | *** | 2,729 | ENSP00000346959 | GO:0005886 | Plasma membrane | + |
| 102 | ***LCP1*** | 1,6195 | 5,8649 | 3,6172 | *** | ** | -0,874 | ENSP00000315757 | GO:0005886 | Plasma membrane | +++ |
| 103 | ***SLC4A2*** | 1,6093 | 6,9301 | 5,2634 | *** | *** | 5,465 | ENSP00000405600 | GO:0005886 | Plasma membrane | ++ |
| 104 | ***FILIP1L*** | 1,6050 | 4,8269 | 3,2432 | ** | ** | -1,876 | ENSP00000346560 | GO:0005886 | Plasma membrane | ++ |
| 105 | ***CCDC81*** | 1,6038 | -0,0690 | 2,5811 | * | * | -3,178 | ENSP00000415528 | GO:0005886 | Plasma membrane | +++ |
| 106 | ***PRICKLE3*** | 1,5849 | 1,9118 | 3,4742 | *** | ** | -0,777 | ENSP00000365494 | GO:0005886 | Plasma membrane | + |
| 107 | ***MRPL42*** | 1,5557 | 5,2662 | 5,9272 | *** | *** | 8,697 | ENSP00000447547 | GO:0005886 | Plasma membrane | +++ |
| 108 | ***NPL*** | 1,5551 | 4,5847 | 3,2022 | ** | ** | -1,965 | ENSP00000258317 | GO:0005886 | Plasma membrane | + |
| 109 | ***PTPN12*** | 1,5504 | 6,6946 | 6,9430 | *** | *** | 13,655 | ENSP00000248594 | GO:0005886 | Plasma membrane | ++ |
| 110 | ***CD9*** | 1,5278 | 8,1540 | 4,0854 | *** | *** | 0,654 | ENSP00000009180 | GO:0005886 | Plasma membrane | ++ |
| 111 | ***VANGL1*** | 1,5256 | 3,4719 | 2,7308 | ** | * | -3,088 | ENSP00000310800 | GO:0005886 | Plasma membrane | ++ |
| 112 | ***BST2*** | 1,5087 | 6,2626 | 2,3938 | * | * | -4,398 | ENSP00000252593 | GO:0005886 | Plasma membrane | ++ |
| 113 | ***ATP10D*** | 1,5041 | 4,7114 | 4,6444 | *** | *** | 3,094 | ENSP00000273859 | GO:0005886 | Plasma membrane | ++ |
| 114 | ***IRAK4*** | 1,4996 | 4,6543 | 4,9822 | *** | *** | 4,505 | ENSP00000390651 | GO:0005886 | Plasma membrane | + |
| 115 | ***FANCG*** | 1,4736 | 4,3882 | 3,9831 | *** | *** | 0,624 | ENSP00000367910 | GO:0005886 | Plasma membrane | + |
| 116 | ***AMDHD1*** | 1,4688 | -0,5063 | 2,6999 | ** | * | -2,902 | ENSP00000266736 | GO:0005886 | Plasma membrane | ++ |
| 117 | ***CD4*** | 1,4542 | 6,2785 | 3,4738 | *** | ** | -1,410 | ENSP00000011653 | GO:0005886 | Plasma membrane | +++ |
| 118 | ***NUP35*** | 1,4504 | 4,1112 | 5,4312 | *** | *** | 6,548 | ENSP00000295119 | GO:0005886 | Plasma membrane | + |
| 119 | ***HN1L*** | 1,4348 | 5,9541 | 5,5789 | *** | *** | 6,951 | ENSP00000248098 | GO:0005886 | Plasma membrane | +++ |
| 120 | ***TRIM38*** | 1,4330 | 3,2996 | 2,9133 | ** | * | -2,591 | ENSP00000230099 | GO:0005886 | Plasma membrane | + |
| 121 | ***LRRC69*** | 1,4242 | -3,8197 | 2,7121 | ** | * | -3,072 | ENSP00000400803 | GO:0005886 | Plasma membrane | ++ |
| 122 | ***PARP14*** | 1,4150 | 5,8816 | 3,3198 | ** | ** | -1,857 | ENSP00000418194 | GO:0005886 | Plasma membrane | + |
| 123 | ***IQGAP1*** | 1,3980 | 7,1794 | 4,0858 | *** | *** | 0,654 | ENSP00000268182 | GO:0005886 | Plasma membrane | + |
| 124 | ***SAAL1*** | 1,3952 | 3,6380 | 5,2958 | *** | *** | 5,995 | ENSP00000432487 | GO:0005886 | Plasma membrane | + |
| 125 | ***RELL1*** | 1,3949 | 4,9526 | 5,9187 | *** | *** | 8,679 | ENSP00000313385 | GO:0005886 | Plasma membrane | ++ |
| 126 | ***BOC*** | 1,3764 | 5,2899 | 3,0991 | ** | ** | -2,437 | ENSP00000347546 | GO:0005886 | Plasma membrane | + |
| 127 | ***MAP4K1*** | 1,3745 | 1,7601 | 2,9931 | ** | ** | -2,208 | ENSP00000380066 | GO:0005886 | Plasma membrane | + |
| 128 | ***FAM129B*** | 1,3377 | 6,7589 | 3,6613 | *** | ** | -0,831 | ENSP00000362409 | GO:0005886 | Plasma membrane | + |
| 129 | ***ABCC4*** | 1,3351 | 4,2389 | 4,0075 | *** | *** | 0,715 | ENSP00000366084 | GO:0005886 | Plasma membrane | + |
| 130 | ***TTC23*** | 1,3206 | 4,3560 | 4,9686 | *** | *** | 4,466 | ENSP00000262074 | GO:0005886 | Plasma membrane | ++ |
| 131 | ***NUBP1*** | 1,2982 | 4,4655 | 6,8267 | *** | *** | 13,256 | ENSP00000283027 | GO:0005886 | Plasma membrane | ++ |
| 132 | ***GSTP1*** | 1,2756 | 7,8904 | 5,0157 | *** | *** | 4,371 | ENSP00000381607 | GO:0005886 | Plasma membrane | + |
| 133 | ***INTS12*** | 1,2720 | 5,0172 | 6,5297 | *** | *** | 11,661 | ENSP00000340737 | GO:0005886 | Plasma membrane | + |
| 134 | ***YES1*** | 1,2672 | 5,9392 | 5,2161 | *** | *** | 5,301 | ENSP00000324740 | GO:0005886 | Plasma membrane | ++ |
| 135 | ***SH3PXD2B*** | 1,2616 | 6,6629 | 3,3435 | ** | ** | -1,860 | ENSP00000309714 | GO:0005886 | Plasma membrane | ++ |
| 136 | ***SLC35F5*** | 1,2604 | 5,1490 | 4,8642 | *** | *** | 3,889 | ENSP00000245680 |  | Plasma membrane | +++ |
| 137 | ***FZD1*** | 1,2414 | 4,9912 | 2,3770 | * | * | -4,283 | ENSP00000287934 | GO:0005886 | Plasma membrane | ++ |
| 138 | ***NT5E*** | 1,2322 | 5,8204 | 2,3824 | * | * | -4,404 | ENSP00000257770 | GO:0005886 | Plasma membrane | ++ |
| 139 | ***ZC3H12A*** | 1,2286 | 2,2973 | 2,5211 | * | * | -3,491 | ENSP00000362174 | GO:0005886 | Plasma membrane | ++ |
| 140 | ***ABCD1*** | 1,2261 | 4,3317 | 3,7564 | *** | *** | -0,206 | ENSP00000218104 | GO:0005886 | Plasma membrane | ++ |
| 141 | ***NCK1*** | 1,2210 | 4,9629 | 5,3671 | *** | *** | 6,097 | ENSP00000288986 | GO:0005886 | Plasma membrane | + |
| 142 | ***PTRF*** | 1,2182 | 7,3271 | 2,6329 | ** | * | -3,879 | ENSP00000349541 | GO:0005886 | Plasma membrane | ++ |
| 143 | ***EFHC1*** | 1,2092 | 4,9935 | 3,3583 | *** | ** | -1,630 | ENSP00000360107 | GO:0005886 | Plasma membrane | ++ |
| 144 | ***C11orf10*** | 1,2050 | 6,1599 | 4,7634 | *** | *** | 3,342 | ENSP00000257262 | GO:0005886 | Plasma membrane | + |
| 145 | ***RDX*** | 1,1963 | 7,9375 | 4,9918 | *** | *** | 4,270 | ENSP00000342830 | GO:0005886 | Plasma membrane | ++ |
| 146 | ***ABHD3*** | 1,1955 | 4,4452 | 4,5380 | *** | *** | 2,667 | ENSP00000289119 | GO:0005886 | Plasma membrane | + |
| 147 | ***RIPK1*** | 1,1859 | 5,4345 | 5,3317 | *** | *** | 5,865 | ENSP00000259808 | GO:0005886 | Plasma membrane | + |
| 148 | ***SLC39A14*** | 1,1845 | 6,7829 | 2,7259 | ** | * | -3,633 | ENSP00000289952 | GO:0005886 | Plasma membrane | ++ |
| 149 | ***HSH2D*** | 1,1811 | -1,1078 | 2,3938 | * | * | -3,653 | ENSP00000253680 | GO:0005886 | Plasma membrane | ++ |
| 150 | ***ITGB8*** | 1,1719 | 7,7626 | 2,6444 | ** | * | -3,847 | ENSP00000222573 | GO:0005886 | Plasma membrane | +++ |
| 151 | ***SNAP23*** | 1,1661 | 5,5962 | 4,7728 | *** | *** | 3,432 | ENSP00000249647 | GO:0005886 | Plasma membrane | +++ |
| 152 | ***KIAA1524*** | 1,1584 | 3,0399 | 2,9398 | ** | * | -2,519 | ENSP00000295746 | GO:0005886 | Plasma membrane | ++ |
| 153 | ***ZNF599*** | 1,1539 | 3,0725 | 5,2705 | *** | *** | 5,918 | ENSP00000333802 | GO:0005886 | Plasma membrane | ++ |
| 154 | ***ITGA7*** | 1,1445 | 7,2472 | 2,4540 | * | * | -4,316 | ENSP00000452120 | GO:0005886 | Plasma membrane | ++ |
| 155 | ***POLE*** | 1,1437 | 4,6706 | 2,6307 | ** | * | -3,635 | ENSP00000322570 | GO:0005886 | Plasma membrane | + |
| 156 | ***SLC43A1*** | 1,1412 | 2,2115 | 3,5364 | *** | ** | -0,638 | ENSP00000278426 | GO:0005886 | Plasma membrane | ++ |
| 157 | ***C8orf33*** | 1,1404 | 7,0785 | 4,5795 | *** | *** | 2,554 | ENSP00000330361 | GO:0005886 | Plasma membrane | + |
| 158 | ***RPIA*** | 1,1390 | 3,8989 | 5,2831 | *** | *** | 5,874 | ENSP00000283646 | GO:0005886 | Plasma membrane | ++ |
| 159 | ***DCBLD2*** | 1,1293 | 5,4574 | 2,3455 | * | * | -4,452 | ENSP00000321573 | GO:0005886 | Plasma membrane | ++ |
| 160 | ***MYBL1*** | 1,1243 | 3,2186 | 2,5225 | * | * | -3,634 | ENSP00000429633 | GO:0005886 | Plasma membrane | + |
| 161 | ***SNX33*** | 1,1056 | 4,6837 | 3,7624 | *** | *** | -0,266 | ENSP00000311427 | GO:0005886 | Plasma membrane | ++ |
| 162 | ***SMAD1*** | 1,1030 | 6,1032 | 4,3580 | *** | *** | 1,716 | ENSP00000305769 | GO:0005886 | Plasma membrane | ++ |
| 163 | ***C3orf38*** | 1,0950 | 5,1350 | 4,7950 | *** | *** | 3,580 | ENSP00000322469 | GO:0005886 | Plasma membrane | ++ |
| 164 | ***SRA1*** | 1,0886 | 5,8261 | 4,6651 | *** | *** | 2,958 | ENSP00000337513 | GO:0005886 | Plasma membrane | + |
| 165 | ***CYP20A1*** | 1,0827 | 4,8366 | 6,5139 | *** | *** | 11,581 | ENSP00000348380 | GO:0005886 | Plasma membrane | ++ |
| 166 | ***ACOT9*** | 1,0688 | 4,8198 | 4,2056 | *** | *** | 1,298 | ENSP00000368605 | GO:0005886 | Plasma membrane | ++ |
| 167 | ***SLC16A1*** | 1,0663 | 7,3612 | 4,4407 | *** | *** | 2,002 | ENSP00000358640 | GO:0005886 | Plasma membrane | ++++ |
| 168 | ***MYO1C*** | 1,0571 | 5,4999 | 3,4782 | *** | ** | -1,351 | ENSP00000352834 | GO:0005886 | Plasma membrane | ++ |
| 169 | ***PDLIM5*** | 1,0396 | 6,7866 | 3,8496 | *** | *** | -0,194 | ENSP00000321746 | GO:0005886 | Plasma membrane | + |
| 170 | ***C14orf93*** | 1,0355 | 3,2748 | 4,2062 | *** | *** | 1,548 | ENSP00000299088 | GO:0005886 | Plasma membrane | +++ |
| 171 | ***IFRD1*** | 1,0319 | 6,4173 | 4,4174 | *** | *** | 1,925 | ENSP00000005558 | GO:0005886 | Plasma membrane | + |
| 172 | ***TMEM43*** | 1,0279 | 6,7688 | 5,7177 | *** | *** | 7,529 | ENSP00000303992 | GO:0005886 | Plasma membrane | ++++ |
| 173 | ***TANK*** | 1,0180 | 5,3915 | 4,4949 | *** | *** | 2,315 | ENSP00000259075 | GO:0005886 | Plasma membrane | + |
| 174 | ***ITGB5*** | 1,0177 | 6,3373 | 2,7221 | ** | * | -3,632 | ENSP00000296181 | GO:0005886 | Plasma membrane | ++ |
| 175 | ***NFKBIA*** | 1,0155 | 6,6993 | 2,5078 | * | * | -4,182 | ENSP00000216797 | GO:0005886 | Plasma membrane | + |
| 176 | ***LMCD1*** | 1,0119 | 4,0959 | 2,5144 | * | * | -3,826 | ENSP00000157600 | GO:0005886 | Plasma membrane | ++ |
| 177 | ***PHACTR4*** | 1,0117 | 5,5508 | 5,3648 | *** | *** | 5,978 | ENSP00000362942 | GO:0005886 | Plasma membrane | ++ |
| 178 | ***FAM101B*** | 1,0103 | 4,7275 | 2,5559 | * | * | -3,851 | ENSP00000331915 | GO:0005886 | Plasma membrane | + |
| 179 | ***UBE2D4*** | 1,0062 | 5,2361 | 3,5187 | *** | ** | -1,190 | ENSP00000222402 | GO:0005886 | Plasma membrane | +++ |
| 180 | ***TULP3*** | 1,0014 | 5,0213 | 4,1513 | *** | *** | 1,044 | ENSP00000380321 | GO:0005886 | Plasma membrane | ++ |
| 181 | ***TMEM97*** | 1,0008 | 5,2206 | 3,5563 | *** | ** | -1,063 | ENSP00000226230 | GO:0005886 | Plasma membrane | ++ |
| 182 | ***SSFA2*** | 0,9975 | 7,6398 | 3,9107 | *** | *** | 0,022 | ENSP00000388731 | GO:0005886 | Plasma membrane | ++ |
| 183 | ***MED28*** | 0,9925 | 5,0271 | 3,7840 | *** | *** | -0,271 | ENSP00000237380 | GO:0005886 | Plasma membrane | + |
| 184 | ***GNAI2*** | 0,9894 | 9,1369 | 5,5761 | *** | *** | 6,906 | ENSP00000312999 | GO:0005886 | Plasma membrane | ++ |
| 185 | ***HOMER3*** | 0,9857 | 5,5388 | 3,9289 | *** | *** | 0,162 | ENSP00000376162 | GO:0005886 | Plasma membrane | + |
| 186 | ***NVL*** | 0,9856 | 4,5498 | 5,4124 | *** | *** | 6,329 | ENSP00000281701 | GO:0005886 | Plasma membrane | + |
| 187 | ***SUCLG2*** | 0,9841 | 5,3485 | 4,0574 | *** | *** | 0,652 | ENSP00000419325 | GO:0005886 | Plasma membrane | ++ |
| 188 | ***RHOA*** | 0,9773 | 9,3900 | 6,4033 | *** | *** | 10,904 | ENSP00000400175 | GO:0005886 | Plasma membrane | + |
| 189 | ***MARCKS*** | 0,9695 | 9,0549 | 3,9165 | *** | *** | 0,080 | ENSP00000357624 | GO:0005886 | Plasma membrane | + |
| 190 | ***NUP43*** | 0,9448 | 5,1225 | 4,4865 | *** | *** | 2,310 | ENSP00000342262 | GO:0005886 | Plasma membrane | ++ |
| 191 | ***RRP8*** | 0,9434 | 4,1204 | 3,8771 | *** | *** | 0,204 | ENSP00000254605 | GO:0005886 | Plasma membrane | + |
| 192 | ***RNF114*** | 0,9364 | 6,7646 | 5,9245 | *** | *** | 8,509 | ENSP00000244061 | GO:0005886 | Plasma membrane | ++ |
| 193 | ***CORO1B*** | 0,9361 | 6,3021 | 5,2566 | *** | *** | 5,434 | ENSP00000340211 | GO:0005886 | Plasma membrane | +++ |
| 194 | ***RRM1*** | 0,9303 | 6,1725 | 3,9977 | *** | *** | 0,353 | ENSP00000300738 | GO:0005886 | Plasma membrane | + |
| 195 | ***C8orf37*** | 0,9224 | 2,8887 | 2,8179 | ** | * | -2,860 | ENSP00000286688 | GO:0005886 | Plasma membrane | ++ |
| 196 | ***SRPRB*** | 0,9211 | 6,3591 | 4,1325 | *** | *** | 0,837 | ENSP00000418401 | GO:0005886 | Plasma membrane | ++ |
| 197 | ***METAP2*** | 0,9197 | 6,9011 | 5,7263 | *** | *** | 7,566 | ENSP00000325312 | GO:0005886 | Plasma membrane | +++ |
| 198 | ***PIGN*** | 0,9191 | 4,6269 | 3,9491 | *** | *** | 0,361 | ENSP00000350263 | GO:0005886 | Plasma membrane | + |
| 199 | ***NFE2L2*** | 0,9162 | 7,1641 | 5,2325 | *** | *** | 5,313 | ENSP00000380252 | GO:0005886 | Plasma membrane | ++ |
| 200 | ***BNIP2*** | 0,9130 | 4,7981 | 4,4684 | *** | *** | 2,283 | ENSP00000267859 | GO:0005886 | Plasma membrane | ++ |
| 201 | ***CDC42EP4*** | 0,9124 | 7,8839 | 2,6316 | ** | * | -3,872 | ENSP00000338258 | GO:0005886 | Plasma membrane | + |
| 202 | ***FHL1*** | 0,9117 | 8,5289 | 2,6700 | ** | * | -3,757 | ENSP00000071281 | GO:0005886 | Plasma membrane | + |
| 203 | ***CCDC59*** | 0,9092 | 4,3422 | 3,2479 | ** | ** | -1,898 | ENSP00000256151 | GO:0005886 | Plasma membrane | + |
| 204 | ***SLC4A7*** | 0,9091 | 4,8005 | 2,6055 | * | * | -3,759 | ENSP00000295736 | GO:0005886 | Plasma membrane | ++ |
| 205 | ***CIB1*** | 0,8974 | 5,9845 | 3,2737 | ** | ** | -2,057 | ENSP00000333873 | GO:0005886 | Plasma membrane | ++ |
| 206 | ***PKN2*** | 0,8884 | 5,6064 | 4,8646 | *** | *** | 3,785 | ENSP00000359552 | GO:0005886 | Plasma membrane | + |
| 207 | ***PPP1CB*** | 0,8783 | 8,9758 | 3,3116 | ** | ** | -1,931 | ENSP00000296122 | GO:0005886 | Plasma membrane | ++ |
| 208 | ***SIPA1*** | 0,8772 | 5,5162 | 3,0666 | ** | ** | -2,632 | ENSP00000377771 | GO:0005886 | Plasma membrane | ++ |
| 209 | ***CRLF3*** | 0,8765 | 3,9754 | 4,0790 | *** | *** | 0,943 | ENSP00000318804 | GO:0005886 | Plasma membrane | +++ |
| 210 | ***HSCB*** | 0,8750 | 3,5640 | 2,4777 | * | * | -3,832 | ENSP00000216027 | GO:0005886 | Plasma membrane | ++ |
| 211 | ***RAB23*** | 0,8732 | 4,8106 | 3,0684 | ** | ** | -2,522 | ENSP00000320413 | GO:0005886 | Plasma membrane | + |
| 212 | ***HPS3*** | 0,8577 | 5,2474 | 3,9608 | *** | *** | 0,299 | ENSP00000296051 | GO:0005886 | Plasma membrane | ++++ |
| 213 | ***FARS2*** | 0,8475 | 3,9739 | 4,4378 | *** | *** | 2,298 | ENSP00000274680 | GO:0005886 | Plasma membrane | ++++ |
| 214 | ***BZW2*** | 0,8458 | 5,9705 | 2,4522 | * | * | -4,289 | ENSP00000258761 | GO:0005886 | Plasma membrane | ++ |
| 215 | ***RAB8A*** | 0,8377 | 6,0837 | 5,0500 | *** | *** | 4,536 | ENSP00000300935 | GO:0005886 | Plasma membrane | ++ |
| 216 | ***CTNND1*** | 0,8342 | 7,7606 | 3,9787 | *** | *** | 0,270 | ENSP00000382004 | GO:0005886 | Plasma membrane | ++ |
| 217 | ***MCAM*** | 0,8310 | 7,1693 | 2,6115 | ** | * | -3,933 | ENSP00000264036 | GO:0005886 | Plasma membrane | ++ |
| 218 | ***ERLIN2*** | 0,8269 | 6,5399 | 4,2865 | *** | *** | 1,409 | ENSP00000276461 | GO:0005886 | Plasma membrane | ++ |
| 219 | ***C17orf62*** | 0,8227 | 6,5173 | 5,0470 | *** | *** | 4,507 | ENSP00000307765 | GO:0005886 | Plasma membrane | +++ |
| 220 | ***RABEPK*** | 0,8178 | 5,2736 | 3,2692 | ** | ** | -2,007 | ENSP00000362639 | GO:0005886 | Plasma membrane | ++ |
| 221 | ***FAM126A*** | 0,8173 | 5,7357 | 2,6533 | ** | * | -3,781 | ENSP00000403396 | GO:0005886 | Plasma membrane | ++ |
| 222 | ***FAM105B*** | 0,8074 | 4,2601 | 4,8673 | *** | *** | 3,976 | ENSP00000284274 | GO:0005886 | Plasma membrane | + |
| 223 | ***PPP1CA*** | 0,8042 | 7,2305 | 3,6680 | *** | ** | -0,821 | ENSP00000326031 | GO:0005886 | Plasma membrane | + |
| 224 | ***HDAC3*** | 0,8037 | 6,0215 | 5,5759 | *** | *** | 6,888 | ENSP00000302967 | GO:0005886 | Plasma membrane | + |
| 225 | ***CAD*** | 0,7948 | 5,7218 | 2,5686 | * | * | -3,993 | ENSP00000264705 | GO:0005886 | Plasma membrane | + |
| 226 | ***EPB41L5*** | 0,7804 | 5,4695 | 3,1870 | ** | ** | -2,283 | ENSP00000263713 | GO:0005886 | Plasma membrane | ++ |
| 227 | ***WASF2*** | 0,7737 | 7,4639 | 3,4119 | *** | ** | -1,655 | ENSP00000396211 | GO:0005886 | Plasma membrane | ++ |
| 228 | ***SETDB1*** | 0,7709 | 5,5705 | 4,3168 | *** | *** | 1,575 | ENSP00000271640 | GO:0005886 | Plasma membrane | + |
| 229 | ***SAMHD1*** | 0,7706 | 5,4816 | 2,3716 | * | * | -4,440 | ENSP00000262878 | GO:0005886 | Plasma membrane | ++ |
| 230 | ***ADCK4*** | 0,7680 | 5,6082 | 3,6059 | *** | ** | -0,977 | ENSP00000315118 | GO:0005886 | Plasma membrane | +++ |
| 231 | ***PDCD2*** | 0,7649 | 5,2469 | 3,1425 | ** | ** | -2,389 | ENSP00000439467 | GO:0005886 | Plasma membrane | ++ |
| 232 | ***PRDX6*** | 0,7624 | 7,9077 | 2,9044 | ** | * | -3,149 | ENSP00000342026 | GO:0005886 | Plasma membrane | ++ |
| 233 | ***ATP6V0A2*** | 0,7618 | 5,0679 | 3,9570 | *** | *** | 0,295 | ENSP00000332247 | GO:0005886 | Plasma membrane | + |
| 234 | ***CD81*** | 0,7596 | 9,8330 | 3,5558 | *** | ** | -1,121 | ENSP00000263645 | GO:0005886 | Plasma membrane | + |
| 235 | ***WDR12*** | 0,7532 | 4,6033 | 3,8592 | *** | *** | 0,025 | ENSP00000261015 | GO:0005886 | Plasma membrane | ++ |
| 236 | ***NSL1*** | 0,7484 | 5,9436 | 3,2077 | ** | ** | -2,263 | ENSP00000355944 | GO:0005886 | Plasma membrane | ++ |
| 237 | ***STAG2*** | 0,7481 | 6,9255 | 3,2695 | ** | ** | -2,103 | ENSP00000218089 | GO:0005886 | Plasma membrane | ++ |
| 238 | ***TDG*** | 0,7431 | 4,2201 | 3,1474 | ** | ** | -2,205 | ENSP00000376611 | GO:0005886 | Plasma membrane | ++ |
| 239 | ***FLAD1*** | 0,7419 | 5,4508 | 4,2139 | *** | *** | 1,193 | ENSP00000292180 | GO:0005886 | Plasma membrane | ++ |
| 240 | ***AVEN*** | 0,7414 | 3,2527 | 2,7360 | ** | * | -3,159 | ENSP00000306822 | GO:0005886 | Plasma membrane | ++ |
| 241 | ***ENAH*** | 0,7405 | 7,3829 | 2,8582 | ** | * | -3,286 | ENSP00000355809 | GO:0005886 | Plasma membrane | ++ |
| 242 | ***PGD*** | 0,7350 | 7,2497 | 3,5870 | *** | ** | -1,090 | ENSP00000270776 | GO:0005886 | Plasma membrane | + |
| 243 | ***EIF2B2*** | 0,7332 | 5,3506 | 3,8396 | *** | *** | -0,158 | ENSP00000266126 | GO:0005886 | Plasma membrane | + |
| 244 | ***SP3*** | 0,7224 | 6,4061 | 4,6120 | *** | *** | 2,689 | ENSP00000310301 | GO:0005886 | Plasma membrane | +++ |
| 245 | ***C1QBP*** | 0,7200 | 6,8770 | 2,9555 | ** | * | -3,022 | ENSP00000225698 | GO:0005886 | Plasma membrane | ++ |
| 246 | ***CCDC97*** | 0,7107 | 5,6898 | 3,8601 | *** | *** | -0,120 | ENSP00000269967 | GO:0005886 | Plasma membrane | + |
| 247 | ***GLE1*** | 0,6997 | 5,4638 | 3,8349 | *** | *** | -0,189 | ENSP00000308622 | GO:0005886 | Plasma membrane | ++ |
| 248 | ***RASSF1*** | 0,6980 | 5,0049 | 3,8656 | *** | *** | -0,028 | ENSP00000349547 | GO:0005886 | Plasma membrane | ++ |
| 249 | ***CAST*** | 0,6949 | 6,7545 | 2,6503 | ** | * | -3,835 | ENSP00000379157 | GO:0005886 | Plasma membrane | +++ |
| 250 | ***SRFBP1*** | 0,6885 | 3,3537 | 3,7509 | *** | *** | -0,138 | ENSP00000341324 | GO:0005886 | Plasma membrane | + |
| 251 | ***ZNF227*** | 0,6880 | 4,5772 | 2,8018 | ** | * | -3,255 | ENSP00000321049 | GO:0005886 | Plasma membrane | +++ |
| 252 | ***EIF5B*** | 0,6875 | 6,8889 | 3,1291 | ** | ** | -2,524 | ENSP00000289371 | GO:0005886 | Plasma membrane | ++ |
| 253 | ***PCBD2*** | 0,6776 | 3,9444 | 2,8335 | ** | * | -3,050 | ENSP00000254908 | GO:0005886 | Plasma membrane | +++ |
| 254 | ***SLC3A2*** | 0,6759 | 8,1311 | 3,2831 | ** | ** | -2,038 | ENSP00000367123 | GO:0005886 | Plasma membrane | ++ |
| 255 | ***KLHL7*** | 0,6681 | 6,3142 | 2,8857 | ** | * | -3,208 | ENSP00000343273 | GO:0005886 | Plasma membrane | + |
| 256 | ***MLH1*** | 0,6605 | 5,6558 | 4,5957 | *** | *** | 2,657 | ENSP00000231790 | GO:0005886 | Plasma membrane | + |
| 257 | ***TBCE*** | 0,6498 | 4,6309 | 3,2642 | ** | ** | -1,947 | ENSP00000355560 | GO:0005886 | Plasma membrane | ++ |
| 258 | ***TAZ*** | 0,6390 | 4,5740 | 2,8573 | ** | * | -3,114 | ENSP00000299328 | GO:0005886 | Plasma membrane | ++ |
| 259 | ***FMNL3*** | 0,6369 | 6,2678 | 2,7410 | ** | * | -3,595 | ENSP00000335655 | GO:0005886 | Plasma membrane | + |
| 260 | ***CFLAR*** | 0,6258 | 4,7412 | 2,7523 | ** | * | -3,423 | ENSP00000312455 | GO:0005886 | Plasma membrane | +++ |
| 261 | ***NUDT16L1*** | 0,6187 | 5,9298 | 2,4361 | * | * | -4,338 | ENSP00000306670 | GO:0005886 | Plasma membrane | ++ |
| 262 | ***PAK1IP1*** | 0,6184 | 4,5278 | 2,8661 | ** | * | -3,081 | ENSP00000368887 | GO:0005886 | Plasma membrane | ++ |
| 263 | ***OSGEP*** | 0,6050 | 4,8000 | 2,5902 | * | * | -3,846 | ENSP00000206542 | GO:0005886 | Plasma membrane | + |
| 264 | ***WDR53*** | 0,5999 | 2,9316 | 2,9783 | ** | ** | -2,477 | ENSP00000328079 | GO:0005886 | Plasma membrane | ++ |
| 265 | ***PSMD9*** | 0,5972 | 5,5041 | 3,0331 | ** | ** | -2,753 | ENSP00000440485 | GO:0005886 | Plasma membrane | ++ |
| 266 | ***MSH6*** | 0,5847 | 5,8643 | 2,4179 | * | * | -4,379 | ENSP00000234420 | GO:0005886 | Plasma membrane | + |
| 267 | ***ATG3*** | 0,5800 | 5,8600 | 3,2790 | ** | ** | -2,051 | ENSP00000283290 | GO:0005886 | Plasma membrane | ++ |
| 268 | ***HERPUD1*** | 0,5795 | 6,7277 | 2,4195 | * | * | -4,397 | ENSP00000409555 | GO:0005886 | Plasma membrane | +++ |
| 269 | ***RIOK2*** | 0,5709 | 4,5881 | 3,8140 | *** | *** | -0,162 | ENSP00000283109 | GO:0005886 | Plasma membrane | ++ |
| 270 | ***FAIM*** | 0,5629 | 3,8412 | 2,5026 | * | * | -3,890 | ENSP00000342805 | GO:0005886 | Plasma membrane | ++ |
| 271 | ***KIAA1430*** | 0,5628 | 5,7365 | 2,6378 | ** | * | -3,838 | ENSP00000409964 | GO:0005886 | Plasma membrane | + |
| 272 | ***DDX23*** | 0,5620 | 7,0371 | 3,3811 | *** | ** | -1,755 | ENSP00000310723 | GO:0005886 | Plasma membrane | + |
| 273 | ***C17orf58*** | 0,5606 | 4,0007 | 2,3373 | * | * | -4,303 | ENSP00000402020 | GO:0005886 | Plasma membrane | ++ |
| 274 | ***MEPCE*** | 0,5010 | 6,3568 | 2,4975 | * | * | -4,210 | ENSP00000308546 | GO:0005886 | Plasma membrane | + |
| 275 | ***STAMBP*** | 0,4903 | 5,7572 | 2,8598 | ** | * | -3,262 | ENSP00000344742 | GO:0005886 | Plasma membrane | ++ |
| 276 | ***TRAF7*** | 0,4801 | 6,5252 | 2,6820 | ** | * | -3,754 | ENSP00000318944 | GO:0005886 | Plasma membrane | ++ |
| 277 | ***C1orf27*** | 0,4736 | 4,8053 | 2,6632 | ** | * | -3,686 | ENSP00000287859 | GO:0005886 | Plasma membrane | ++ |
| 278 | ***CTNNB1*** | 0,4642 | 8,8793 | 2,9094 | ** | * | -3,097 | ENSP00000344456 | GO:0005886 | Plasma membrane | +++ |
| 279 | ***C16orf88*** | 0,4633 | 4,2277 | 2,3004 | * | * | -4,454 | ENSP00000219837 | GO:0005886 | Plasma membrane | + |
| 280 | ***ZNF655*** | 0,4596 | 6,2709 | 2,5235 | * | * | -4,147 | ENSP00000393876 | GO:0005886 | Plasma membrane | +++ |
| 281 | ***GOLPH3L*** | 0,4560 | 4,7326 | 2,4049 | * | * | -4,301 | ENSP00000271732 | GO:0005886 | Plasma membrane | ++ |
| 282 | ***PIP5K1A*** | 0,4416 | 6,1430 | 2,7091 | ** | * | -3,679 | ENSP00000357883 | GO:0005886 | Plasma membrane | ++ |
| 283 | ***PDAP1*** | 0,4218 | 7,1045 | 2,3207 | * | * | -4,619 | ENSP00000222968 | GO:0005886 | Plasma membrane | ++ |
| 284 | ***CCT3*** | 0,4082 | 8,1023 | 2,4002 | * | * | -4,412 | ENSP00000295688 | GO:0005886 | Plasma membrane | ++ |
| 285 | ***NAB1*** | 0,4074 | 5,7187 | 2,6260 | ** | * | -3,875 | ENSP00000336894 | GO:0005886 | Plasma membrane | + |
| 286 | ***XRCC5*** | 0,4059 | 8,1966 | 2,9150 | ** | * | -3,102 | ENSP00000375977 | GO:0005886 | Plasma membrane | + |
| 287 | ***MARCH7*** | 0,3661 | 6,2131 | 2,8699 | ** | * | -3,255 | ENSP00000259050 | GO:0005886 | Plasma membrane | ++ |

**Supplementary Table S1.** Differentially expressed genes in glioblastoma samples. RNAseq analysis of 142 glioblastoma samples vs. 5 healthy brain samples, ranked by differential expression, Log_2_FC; The Cancer Genome Atlas available from https://www.cancer.gov/tcga [26]. The selection of genes encoding proteins localized at the plasma membrane (GO:0005886) led to 287 potential glioblastoma biomarker candidates (Human Protein Atlas available from http://www.proteinatlas.org [35]). ***p<0.001; **p<0.01; *p<0.05. Evidence of plasma membrane location strong (++++); moderate (+++); weak (++); very weak (+).

**a**

| **No.** | **Start** | **End** | **Peptide** |
| --- | --- | --- | --- |
| 1 | 3 | 32 | ALCGSGELGSKFWDSNLSVHTENPDLTPCF |
| 2 | 96 | 105 | VHGRAPAPVF |
| 3 | 128 | 134 | LQGVQSS |
| 4 | 160 | 170 | KAEGEISDPFR |
| 5 | 191 | 213 | REKPPFFSAKNVDPNPYPETSAG |
| 6 | 231 | 251 | YRHPLEEKDLWSLKEEDRSQM |
| 7 | 259 | 299 | AWRKQEKQTARHKASAAPGKNASGEDEVLLGARPRPRKPSF |
| 8 | 341 | 347 | NPMAPSW |
| 9 | 380 | 381 | KF |
| 10 | 399 | 407 | SVKRASTVG |
| 11 | 415 | 415 | V |
| 12 | 418 | 418 | Q |
| 13 | 420 | 420 | F |
| 14 | 448 | 453 | LGPSVL |
| 15 | 468 | 470 | AVA |
| 16 | 481 | 485 | MKLKD |
| 17 | 506 | 506 | W |
| 18 | 508 | 520 | PSFLKQVEGIRQG |
| 19 | 530 | 534 | YLHTT |
| 20 | 537 | 538 | FT |
| 21 | 557 | 569 | VDPNNVLDAEKAF |
| 22 | 590 | 598 | SNLTQASVS |
| 23 | 606 | 658 | LSQEELDPQSVERKTISPGYAITIHSGTFTWAQDLPPTLHSLDIQVPKGALVA |
| 24 | 660 | 670 | VGPVGCGKSSL |
| 25 | 678 | 700 | MEKLEGKVHMKGSVAYVPQQAWI |
| 26 | 704 | 705 | TL |
| 27 | 714 | 724 | ALNPKRYQQTL |
| 28 | 735 | 760 | MLPGGDQTEIGEKGINLSGGQRQRVS |
| 29 | 764 | 768 | AVYSD |
| 30 | 777 | 785 | PLSAVDSHV |
| 31 | 793 | 802 | VIGPEGVLAG |
| 32 | 813 | 816 | SFLP |
| 33 | 827 | 844 | GQVSEMGPYPALLQRNGS |
| 34 | 851 | 878 | NYAPDEDQGHLEDSWTALEGAEDKEALL |
| 35 | 882 | 898 | TLSNHTDLTDNDPVTYV |
| 36 | 909 | 953 | ALSSDGEGQGRPVPRRHLGPSEKVQVTEAKADGALTQEEKAAIGT |
| 37 | 965 | 965 | A |
| 38 | 981 | 987 | QSAAAIG |
| 39 | 993 | 1010 | SAWTNDAMADSRQNNTSL |
| 40 | 1033 | 1040 | MAAGGIQA |
| 41 | 1053 | 1067 | IRSPQSFFDTTPSGR |
| 42 | 1077 | 1077 | Y |
| 43 | 1129 | 1186 | YAATSRQLKRLESVSRSPIYSHFSETVTGASVIRAYNRSRDFEIISDTKVDANQRSCY |
| 44 | 1218 | 1227 | RSSLNPGLVG |
| 45 | 1249 | 1253 | DLESN |
| 46 | 1255 | 1255 | V |
| 47 | 1259 | 1301 | RVKEYSKTETEAPWVVEGSRPPEGWPPRGEVEFRNYSVRYRPG |
| 48 | 1313 | 1331 | VHGGEKVGIVGRTGAGKSS |
| 49 | 1342 | 1350 | AAKGEIRID |
| 50 | 1352 | 1354 | LNV |
| 51 | 1357 | 1357 | I |
| 52 | 1367 | 1376 | TIIPQDPILF |
| 53 | 1381 | 1396 | RMNLDPFGSYSEEDIW |
| 54 | 1407 | 1429 | FVSSQPAGLDFQCSEGGENLSVG |
| 55 | 1452 | 1465 | ATAAIDLETDNLIQ |
| 56 | 1469 | 1473 | RTQFD |
| 57 | 1500 | 1511 | VVAEFDSPANLI |

**b**

| **Length** | **Start** | **End** | **Peptide** | **Location** |
| --- | --- | --- | --- | --- |
| **30** | **3** | **32** | **ALCGSGELGSKFWDSNLSVHTENPDLTPCF** | Extracellular |
| 28 | 851 | 878 | NYAPDEDQGHLEDSWTALEGAEDKEALL | Intracellular |
| 26 | 735 | 760 | MLPGGDQTEIGEKGINLSGGQRQRVS | Intracellular |
| 23 | 191 | 213 | REKPPFFSAKNVDPNPYPETSAG | Intracellular |
| 23 | 678 | 700 | MEKLEGKVHMKGSVAYVPQQAWI | Intracellular |
| 23 | 1407 | 1429 | FVSSQPAGLDFQCSEGGENLSVG | Intracellular |
| 21 | 231 | 251 | YRHPLEEKDLWSLKEEDRSQM | Intracellular |
| 19 | 1313 | 1331 | VHGGEKVGIVGRTGAGKSS | Intracellular |
| 18 | 827 | 844 | GQVSEMGPYPALLQRNGS | Intracellular |
| **18** | **993** | **1010** | **SAWTNDAMADSRQNNTSL** | Extracellular |
| 17 | 882 | 898 | TLSNHTDLTDNDPVTYV | Intracellular |
| 16 | 1381 | 1396 | RMNLDPFGSYSEEDIW | Intracellular |
| 15 | 1053 | 1067 | IRSPQSFFDTTPSGR | Intracellular |
| 14 | 1452 | 1465 | ATAAIDLETDNLIQ | Intracellular |
| 13 | 508 | 520 | PSFLKQVEGIRQG | Intracellular |
| **13** | **557** | **569** | **VDPNNVLDAEKAF** | **Extracellular** |
| 12 | 1500 | 1511 | VVAEFDSPANLI | Intracellular |
| **11** | **160** | **170** | **KAEGEISDPFR** | Extracellular |
| 11 | 660 | 670 | VGPVGCGKSSL | Intracellular |
| 11 | 714 | 724 | ALNPKRYQQTL | Intracellular |

**Supplementary Table S2.** Candidate immunogenic epitopes within the ABCC3 protein sequence. **(a)** The selection of linear (sequential) epitopes led to 57 potential immunogenic epitopes (BepiPred analysis, IEDB available from <https://www.iedb.org/> [31]. **(b)** The further selection of epitopes with a length in accordance with that characteristic of B-cell epitopes (11-30 amino acids), and extracellular surface location led to 4 immunogenic-like peptides (ABCC3-A, amino acids 3-32; ABCC3-K, amino acids 160-170; ABCC3-V, amino acids 557-569; and ABCC3-L, amino acids 993-1,010) which could serve for a peptide-based strategy for panning of the phage-display library of nanobodies.

| **Signal 1,2** | | | | | | | | | | | | | | | | | | | | | | | |
| --- | --- | --- | --- | --- | --- | --- | --- | --- | --- | --- | --- | --- | --- | --- | --- | --- | --- | --- | --- | --- | --- | --- | --- |
| **A** | 26 | 35 | 58 | 98 | 102 | 106 | 132 | 134 | 146 | 150 | 153 | 171 | 182 | 204 | 207 | 218 |  |  |  |  |  |  |  |
| **K** | 5 | 6 | 14 | 20 | 29 | 31 | 32 | 33 | 35 | 36 | 38 | 41 | 46 | 69 | 87 | 88 | 90 | 91 | 92 | 93 | 95 | 97 | 99 |
|  | 100 | 101 | 109 | 111 | 116 | 117 | 122 | 125 | 131 | 136 | 137 | 138 | 139 | 141 | 142 | 143 | 149 | 156 | 158 | 170 | 175 | 182 | 184 |
|  | 193 | 198 | 199 | 200 | 202 | 203 | 204 | 206 | 212 | 214 | 218 | 233 | 235 | 238 |  |  |  |  |  |  |  |  |  |
| **L** | 11 | 29 | 32 | 35 | 37 | 46 | 52 | 53 | 60 | 65 | 70 | 123 | 152 | 165 | 186 | 196 | 197 | 198 | 199 | 205 | 218 | 225 | 237 |

| **Signal >1.5** | | | | | |
| --- | --- | --- | --- | --- | --- |
| **A** | 42 | 49 | 92 | 213 | 237 |
| **K** | 2 | 39 | 145 | 229 |  |
| **L** | 51 | 91 | 115 |  |  |

**Supplementary Table S3.** Screening of nanobodies of positive clones targeting immunogenic epitopes of ABCC3 by ELISA. Potential specific clones showing 1.5 higher signal in the peptide-coated well than the negative were selected to further validate the immunoreactivity against ABCC3 expressing cells.
